# Supplementary material for: Unlocking plant health survey data: An approach to quantify the sensitivity and specificity of visual inspections
Source: PLoS Comput Biol. 2025 Nov 20;21(11):e1012957. doi: 10.1371/journal.pcbi.1012957 (PMC12671890; doi:10.1371/journal.pcbi.1012957)
Supplement: S1 File — (PDF) [file pcbi.1012957.s001.pdf]

Volunteer Background Questions and Responses

(1) On average, how much time do you spend with trees or in woodlands?

	Number of participants
I spend time near trees or in woodlands most days	13
I spend time near trees or in woodlands 2 or 3 times a week	9
I spend time near trees or in woodlands a few times a month	1
I rarely spend time near trees or in woodlands	0
I hardly ever spend time near trees or in woodlands	0

(2) Have you ever worked as a professional tree surveyor (in any sector for example: Forestry, Arboriculture or Local authority)?

	Number of participants
Yes	9
No	14

(3) How confidently do you believe that you could tell the difference between an oak, a beech, an ash, an alder and a field maple?

	Number of participants
Completely confident	17
Fairly confident	3
Somewhat confident	1
Slightly confident	1
Not confident at all	1

(4) Prior to this project, how would you rate your knowledge of Acute Oak Decline (AOD)?

	Number of participants
Completely knowledgeable	2
Fairly knowledgeable	4
Somewhat knowledgeable	8
Slightly knowledgeable	6
Not knowledgeable at all	3

(5) Please state which of the following you consider to be True or False*:

	Number of participants	
	**True**	**False**
Before this project, I had never heard of AOD	4	19
Before this project, I was aware of AOD, but not the symptoms	7	14
Before this project, I was aware of the symptoms of AOD, but had not seen any of them on an oak	6	14
Before this project, I had seen bleeds on trees before (on any tree species)	20	2
Before this project, I had seen Agrilus emergence holes before (on any tree species)	13	8
Before this project, I had seen both bleeds and emergence holes before (on the same oak tree)	7	14

*Please note that some respondents' answers to these questions were not consistent, so may have misinterpreted wording of the questions.

(6) Prior to participation in this project, how would you rate your ability to recognise bleeds on trees?

	Number of participants
Completely confident	7
Fairly confident	7
Somewhat confident	5
Slightly confident	1
Not confident at all	1

(7) Prior to participation in this project, how would you rate your ability to recognise Agrilus emergence holes on trees?

	Number of participants
Completely confident	2
Fairly confident	9
Somewhat confident	2
Slightly confident	3
Not confident at all	5

(8) After participation in this project, how would you rate your knowledge of Acute Oak Decline (AOD)?

	Number of participants
Completely knowledgeable	3
Fairly knowledgeable	14
Somewhat knowledgeable	3
Slightly knowledgeable	0
Not knowledgeable at all	0

(9) After participation in this project, how would you rate your ability to recognise bleeds on trees?

	Number of participants
Completely confident	9
Fairly confident	10
Somewhat confident	1
Slightly confident	0
Not confident at all	0

(10) After participation in this project, how would you rate your ability to recognise Agrilus emergence holes on trees?

	Number of participants
Completely confident	7
Fairly confident	11
Somewhat confident	2
Slightly confident	0
Not confident at all	0

Volunteer Training

Training of volunteers for the survey days involved a 30 minute webinar which outlined the project rationale (approximately 10 minutes), the history of AOD, along with the causes and symptoms (20 minutes). On the day of the assessment, all volunteers were involved in a 15 minute discussion on the history and distribution of AOD, then followed by a 30 minute discussion of AOD symptoms using an AOD field, and an inspection of three trees displaying AOD symptoms.
