# Supplementary material for: Unlocking plant health survey data: An approach to quantify the sensitivity and specificity of visual inspections
Source: PLoS Comput Biol. 2025 Nov 20;21(11):e1012957. doi: 10.1371/journal.pcbi.1012957 (PMC12671890; doi:10.1371/journal.pcbi.1012957)
Supplement: S4 File — (PDF) [file pcbi.1012957.s004.pdf]

Covariance parameter details

The covariance parameter for tests on disease positive hosts, $CovD^+$, is defined as:

$CovD^+ = Se_{12} - Se_1 Se_2$

Where:

$Se_1 = Probability(Symptom\ 1\ positive \mid Diseased\ host)$,

$Se_2 = Probability(Symptom\ 2\ positive \mid Diseased\ host)$,

$Se_{12} = Probability(Symptom\ 1\ positive, Symtpom\ 2\ positive \mid Diseased\ host)$.

This means that for tests of disease positives whose outcomes are independent of another $CovD^+$ would have a value of 0, i.e. the probability both tests positive and tree infected minus probability that both tests are positive given an infected tree if both tests are independent.

This similarly relates to the covariance parameter for tests on disease negatives hosts, $CovD^-$, which is defined as:

$CovD^- = Sp_{12} - Sp_1 Sp_2$

Where:

$Sp_1 = Probability(Symptom\ 1\ negative \mid Disease\ free\ host)$,

$Sp_2 = Probability(\ Symtpom\ 2\ negative \mid Disease\ free\ host)$,

$Sp_{12} = Probability(Symptom\ 1\ negative, Symtpom\ 2\ negative \mid Disease\ free\ host)$.

This means that for tests of disease negatives whose outcomes are independent of another $CovD^-$ would have a value of 0, i.e. the probability both tests negative and tree infected minus probability that both tests are negative given an infected tree if both tests are independent.

We can also derive the maximum and minimum possible values for the parameters $CovD^+$and $CovD^-$.

For $CovD^+$ the maximum possible value can be defined as:

$Max\ covD^+: \min(Se_1, Se_2) - Se_1 Se_2$

This represents that it is not possible for the combined sensitivity of tests, Se_{12}, to be greater than the minimum sensitivity, Se, value of one of the tests.

The minimum possible value can be defined as:

$Min\ covD^+: (Se_1 - 1)(1 - Se_2)$

This represents that it is not possible to have a combined sensitivity of tests, Se_{12}, that is less than the minimum value if both test sensitivity were always negatively related, i.e. the negative of Se_1Se_2.

This is similarly expressed for tests of disease negatives as:

$Max\ covD^{-}: \min(Sp_1, Sp_2) - Sp_1Sp_2$

Represents that it is not possible for the combined specificity of tests, Sp_{12}, to be greater than the minimum specificity, Sp, value of one of the tests.

$Min\ covD^{-}: (Sp_1 - 1)(1 - Sp_2)$

Represents that it is not possible to have a combined specificity of tests, Sp_{12}, that is less than the minimum value if both test specificity were always negatively related, i.e. the negative of Sp_1Sp_2.

Practically, we can therefore assume that tests with related sources of false negatives will lead to non-independence of tests for disease positives, whilst related sources of false positives will lead to non-independence of tests for disease negatives. The example provided in the manuscript of two tests that can be expected to have non-independence are detection of a pest from a necrotic lesion on a host using either an ELISA test for a pest antigen, or qPCR test for pest DNA. Both tests are examining whether pest material is present within a localised symptom on a host, and can therefore be expected to have related false negatives for detection of the pest because they are examining a comparable biological process (i.e. the pest is present and presenting necrotic lesions on a host).

Obtaining independence between test errors can be achieved if the tests represent biologically separate processes.

Examples of test independence provided in the text are between examining the presence of soil samples for the presence of a pest (e.g. soil baiting for *Phytophthora* spp.), and visual inspection for a *Phytophthora* spp. induced stem bleed on a host. These diagnostic tests are sufficiently biologically separate and thus associated with different errors:

False negatives associated with soil sampling: Low concentration of *Phytophthora* in the soil resulting in poor sampling efficacy, incorrect soil sample storage, absence of *Phytophthora* in the soil at a given sample location from random error due to limit on the amount of soil taken.

False positives associated with soil sampling: Could occur from cross-contamination in the sampling process, or cross-reaction in a PCR with a related species that is present in the soil but not pathogenic.

False negatives associated with visual inspection for stem bleeds: Tree infected but not yet symptomatic and failure of observer to witness symptom.

False positives associated with visual inspection for stem bleeds: Another causal agent of a bleed on a stem.

An example of test independence provided in the text between visual symptoms given in the text is between the detection of canopy dieback, and the detection of stem bleeds on a tree. Both of these symptoms can be caused by different alternative agents (e.g. canopy dieback: root compression, drought stress, root rotting pathogen; Stem bleeds: other potentially pathogenic species e.g. a range of *Phytophthora* spp. physical wounding, bacteria such as *Pseudomonas* spp.).
