# Supplementary figures and images for: Unlocking plant health survey data: An approach to quantify the sensitivity and specificity of visual inspections
Source: PLoS Comput Biol. 2025 Nov 20;21(11):e1012957. doi: 10.1371/journal.pcbi.1012957 (PMC12671890; doi:10.1371/journal.pcbi.1012957)

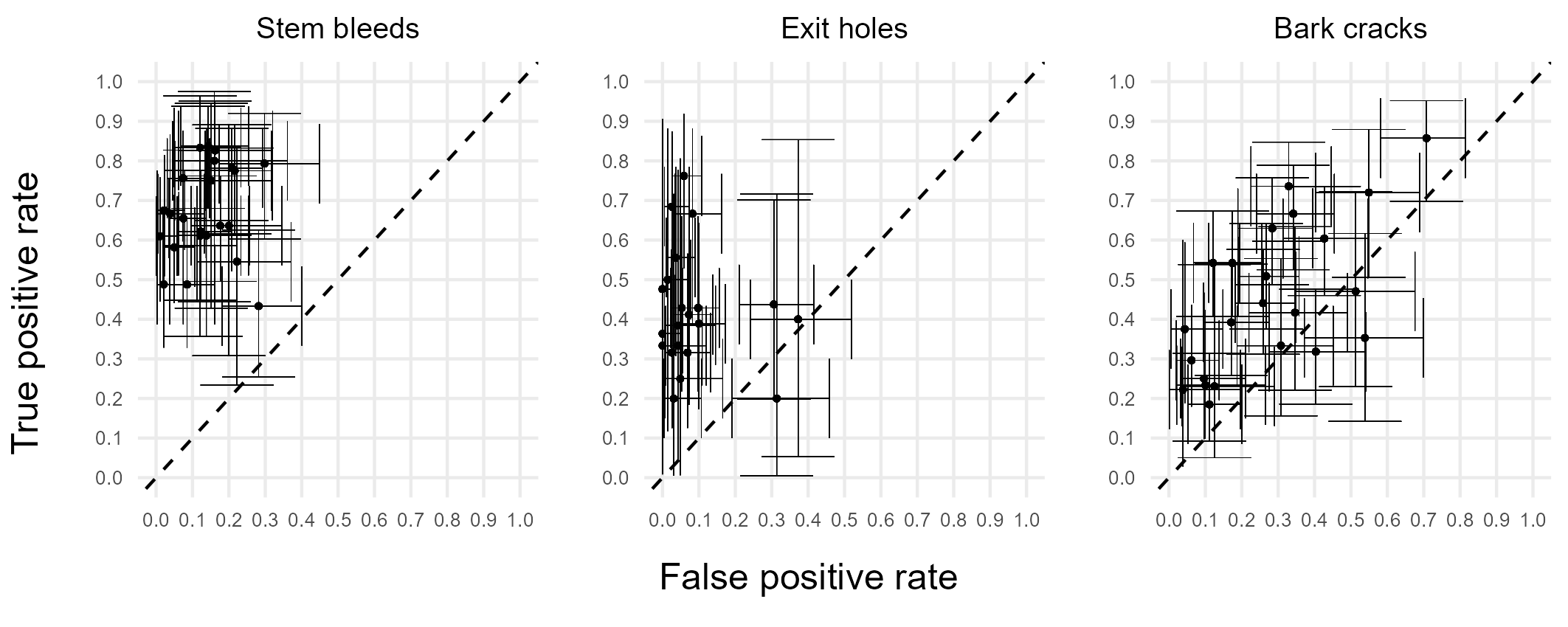

Supplement: S1 Fig — The presence/ absence of bleeds/ weeping patches on the main stem, Agrilus beetle exit holes and cracks between bark plates were assessed by 23 surveyors on up to 175 oak trees. True positive rate and false positive rate were calculated using expert data as a ‘gold-standard’. Error bars represent 95% confidence intervals. (TIF) [file pcbi.1012957.s005.tif]

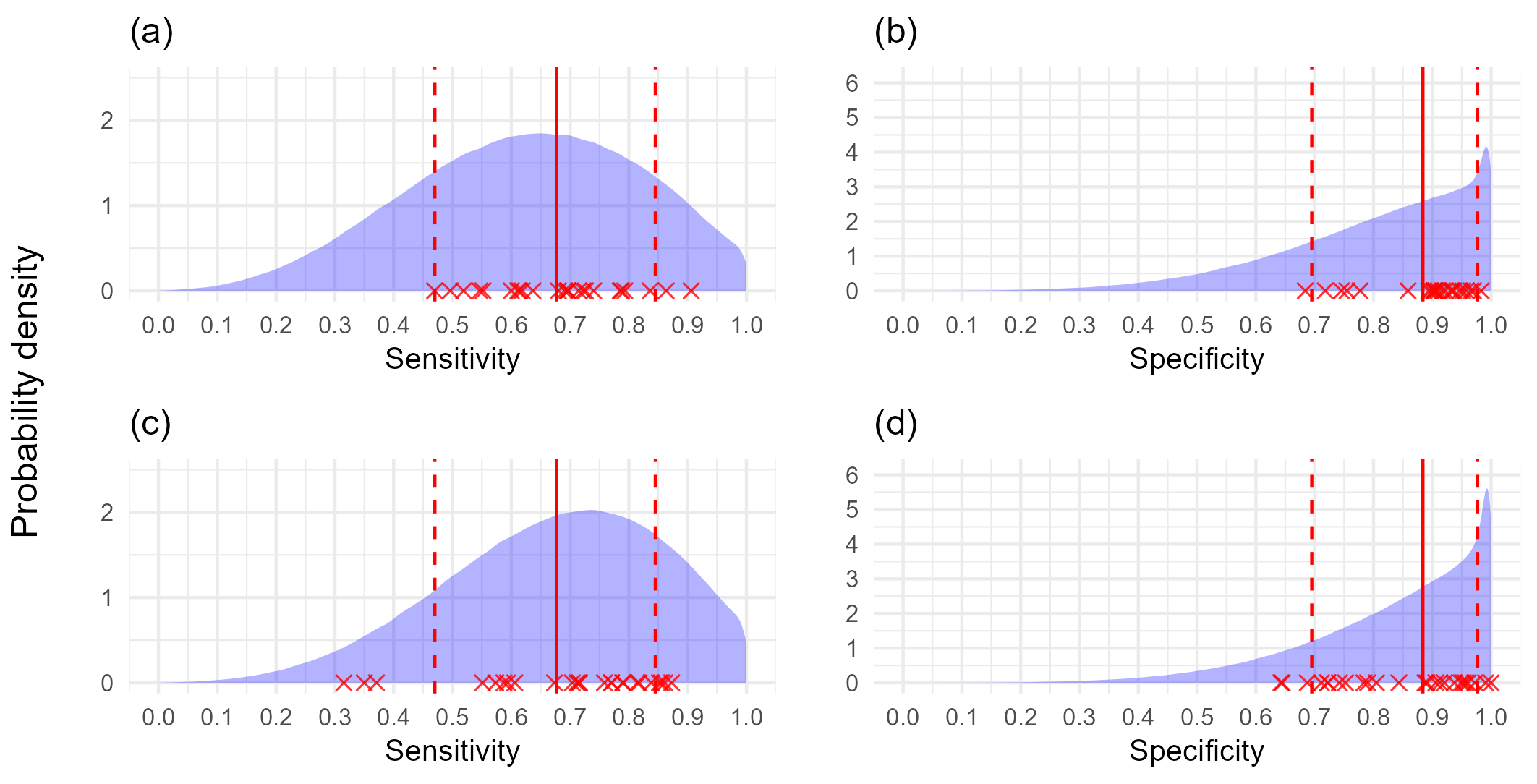

Supplement: S2 Fig — Probability density of estimated sensitivity and specificity of surveyors using poor sensitivity and specificity prior distributions for symptom one, and very good sensitivity and specificity prior distributions for symptom two with reliable prior knowledge of true disease prevalence with (a, b) no covariance model with no simulated covariance, (c, d) covariance model with simulated covariance. In both instances 80 trees each were assessed in higher and lower true disease prevalence locations by 25 surveyors. Red crosses represent true surveyor sensitivity and specificity. Solid red line represents the 50th percentile (median), dotted red lines represent the 5th and 95th percentiles of the distributions surveyor sensitivity and specificity values were generated from. (TIF) [file pcbi.1012957.s006.tif]

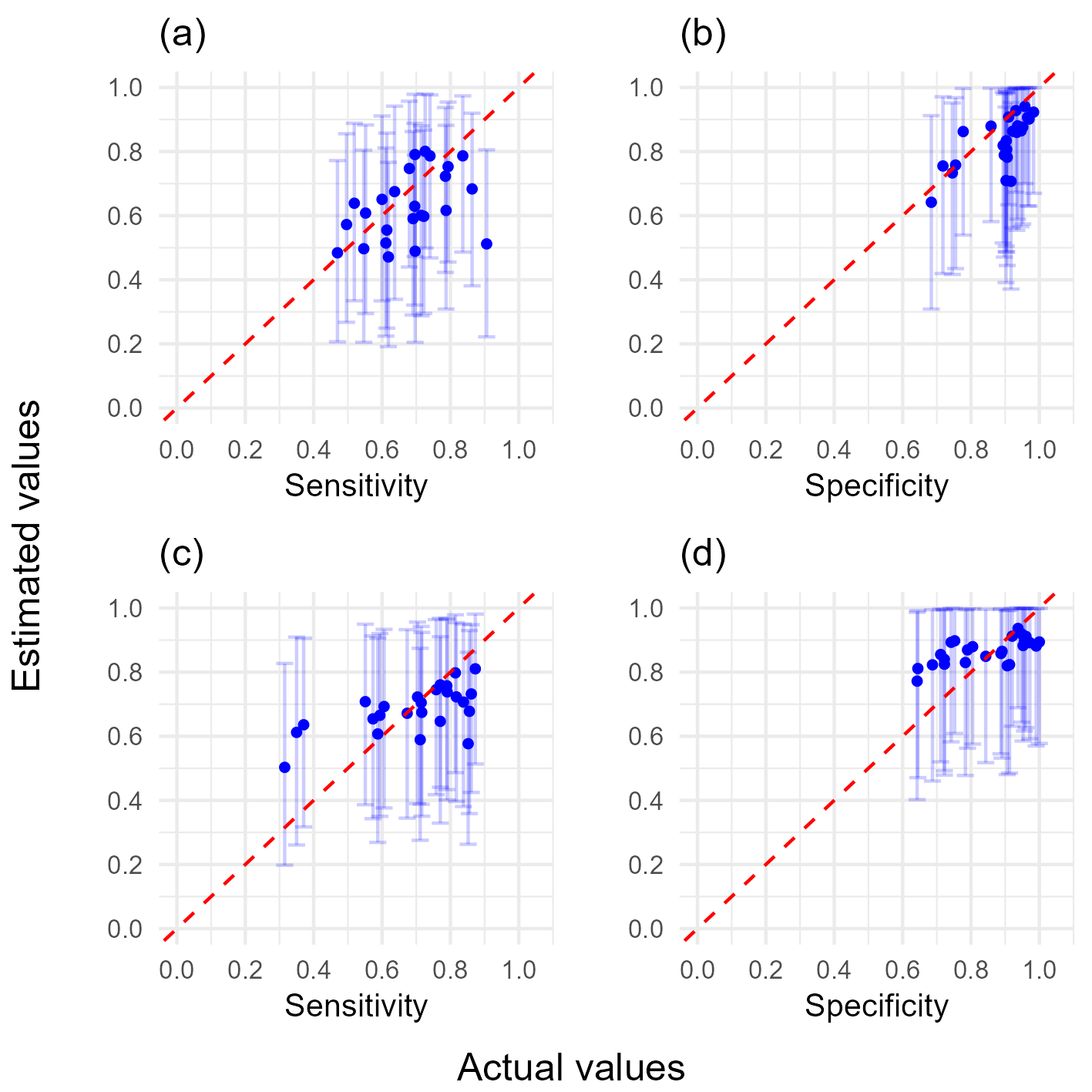

Supplement: S3 Fig — Poor sensitivity and specificity prior distributions for symptom one, and very good sensitivity and specificity prior distributions for symptom two were used with reliable prior knowledge of true disease prevalence with (a, b) no covariance model with no simulated covariance, (c, d) covariance model with simulated covariance. In both instances 80 trees each were assessed in higher and lower true disease prevalence locations. Error bars represent 95% confidence intervals, and dashed line represents perfect agreement between estimated values and actual values. (TIF) [file pcbi.1012957.s007.tif]

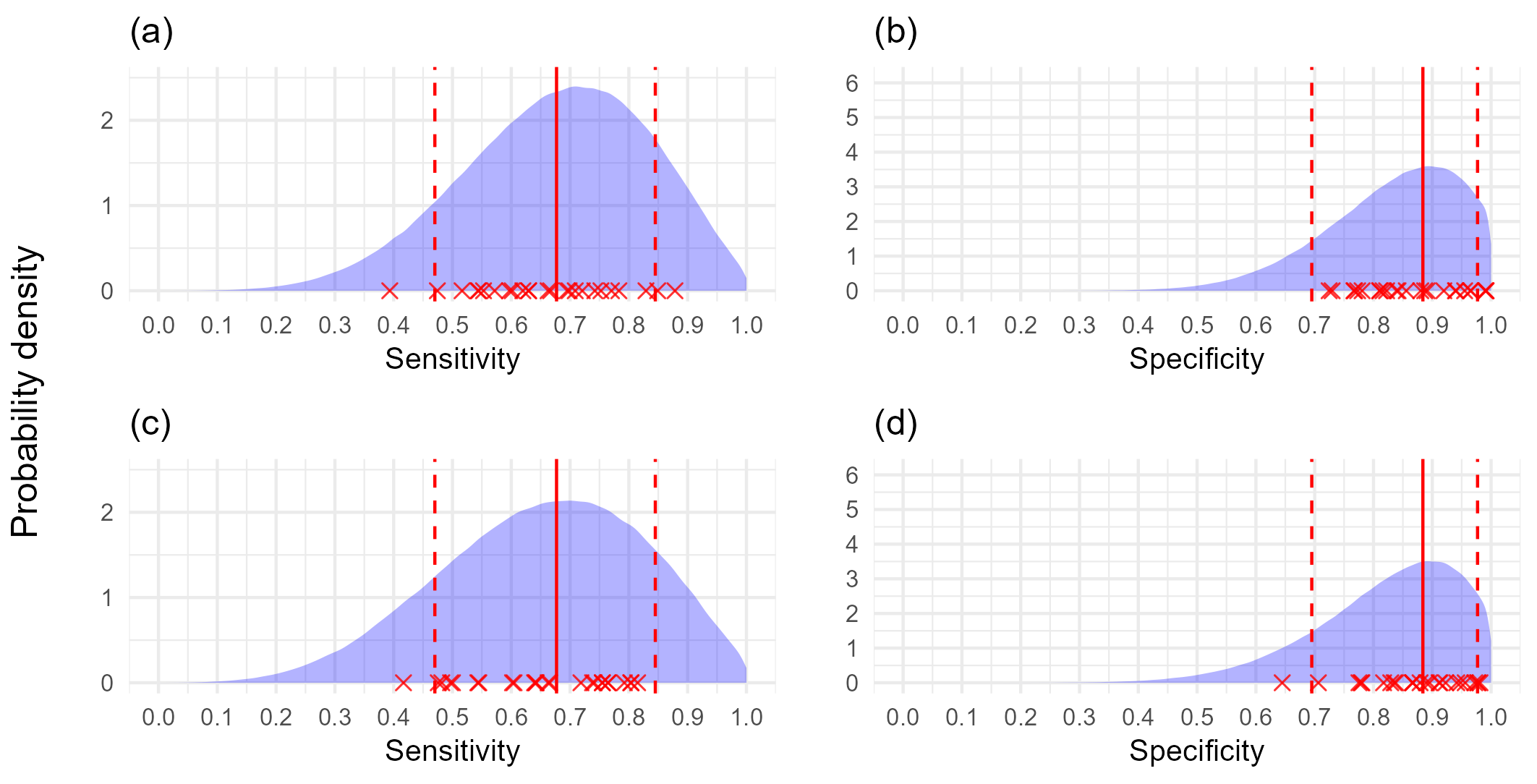

Supplement: S4 Fig — Probability density of estimated sensitivity and specificity of surveyors using good sensitivity and specificity prior distributions with reliable prior knowledge of true disease prevalence with (a, b) no covariance model with no simulated covariance, (c, d) covariance model with simulated covariance. In both instances 80 trees each were assessed in higher and lower true disease prevalence locations by 25 surveyors. Red crosses represent true surveyor sensitivity and specificity. Solid red line represents the 50th percentile (median), dotted red lines represent the 5th and 95th percentiles of the distributions surveyor sensitivity and specificity values were generated from. (TIF) [file pcbi.1012957.s008.tif]

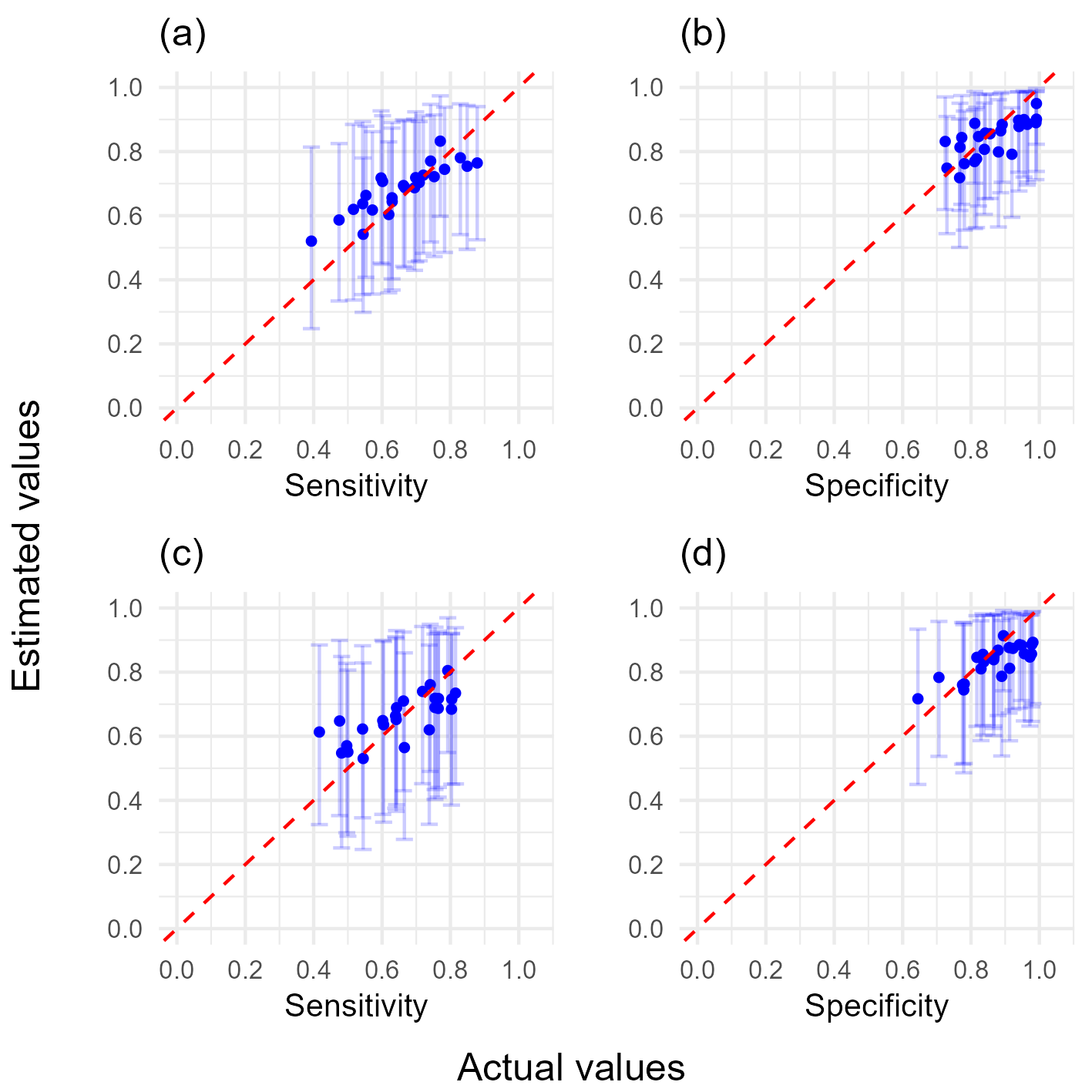

Supplement: S5 Fig — Good sensitivity and specificity prior distributions were used with reliable prior knowledge of true disease prevalence with (a, b) no covariance model with no simulated covariance, (c, d) covariance model with simulated covariance. In both instances 80 trees each were assessed in higher and lower true disease prevalence locations. Error bars represent 95% confidence intervals, and dashed line represents perfect agreement between estimated values and actual values. (TIF) [file pcbi.1012957.s009.tif]

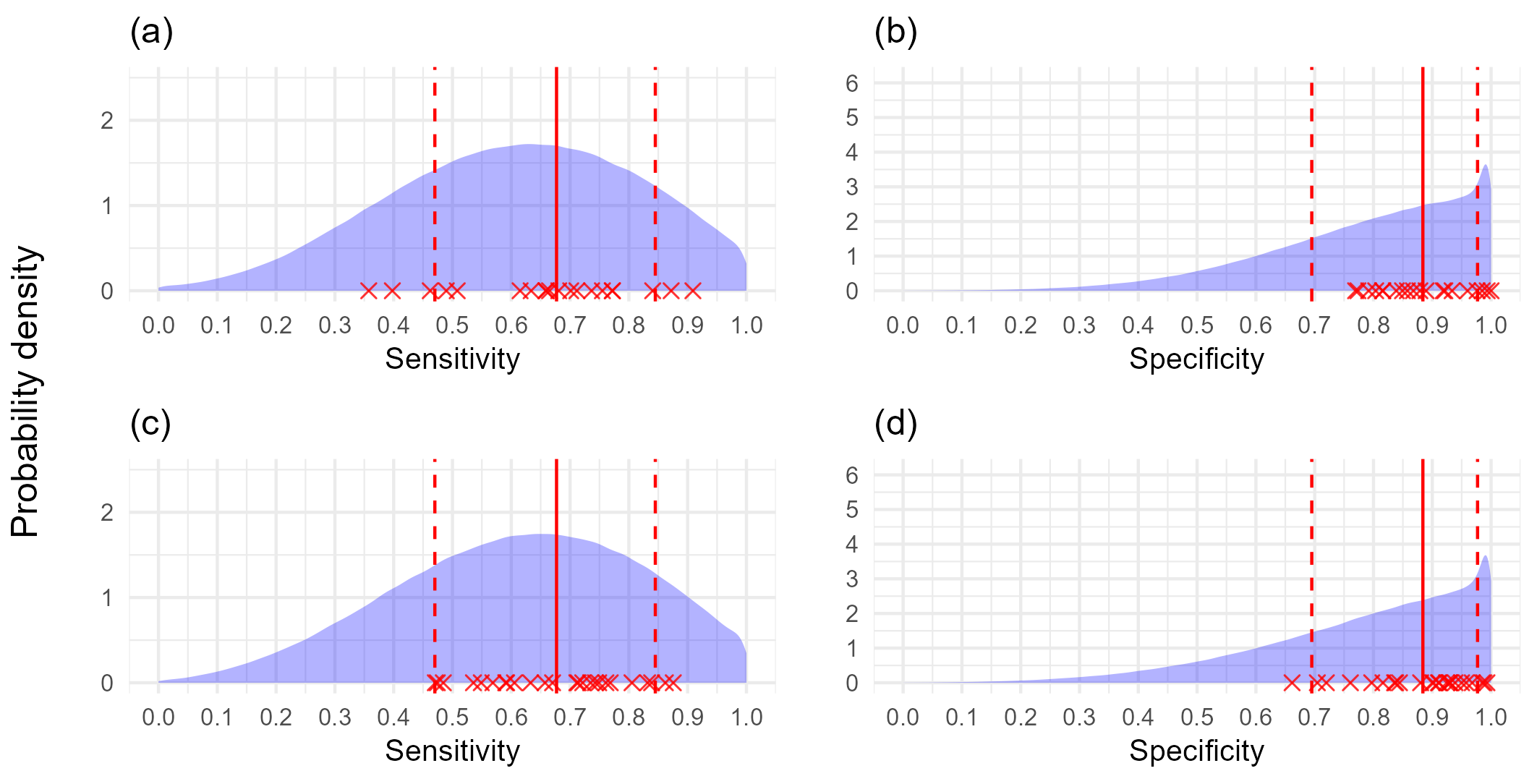

Supplement: S6 Fig — Probability density of estimated sensitivity and specificity of surveyors using poor sensitivity and specificity prior distributions with reliable prior knowledge of true disease prevalence with (a, b) no covariance model with no simulated covariance, (c, d) covariance model with simulated covariance. In both instances 100 trees each were assessed in higher and lower true disease prevalence locations by 25 surveyors, but 5/25 models did not adequately converge for the no covariance model and were discarded. Red crosses represent true surveyor sensitivity and specificity. Solid red line represents the 50th percentile (median), dotted red lines represent the 5th and 95th percentiles of the distributions surveyor sensitivity and specificity values were generated from. (TIF) [file pcbi.1012957.s010.tif]

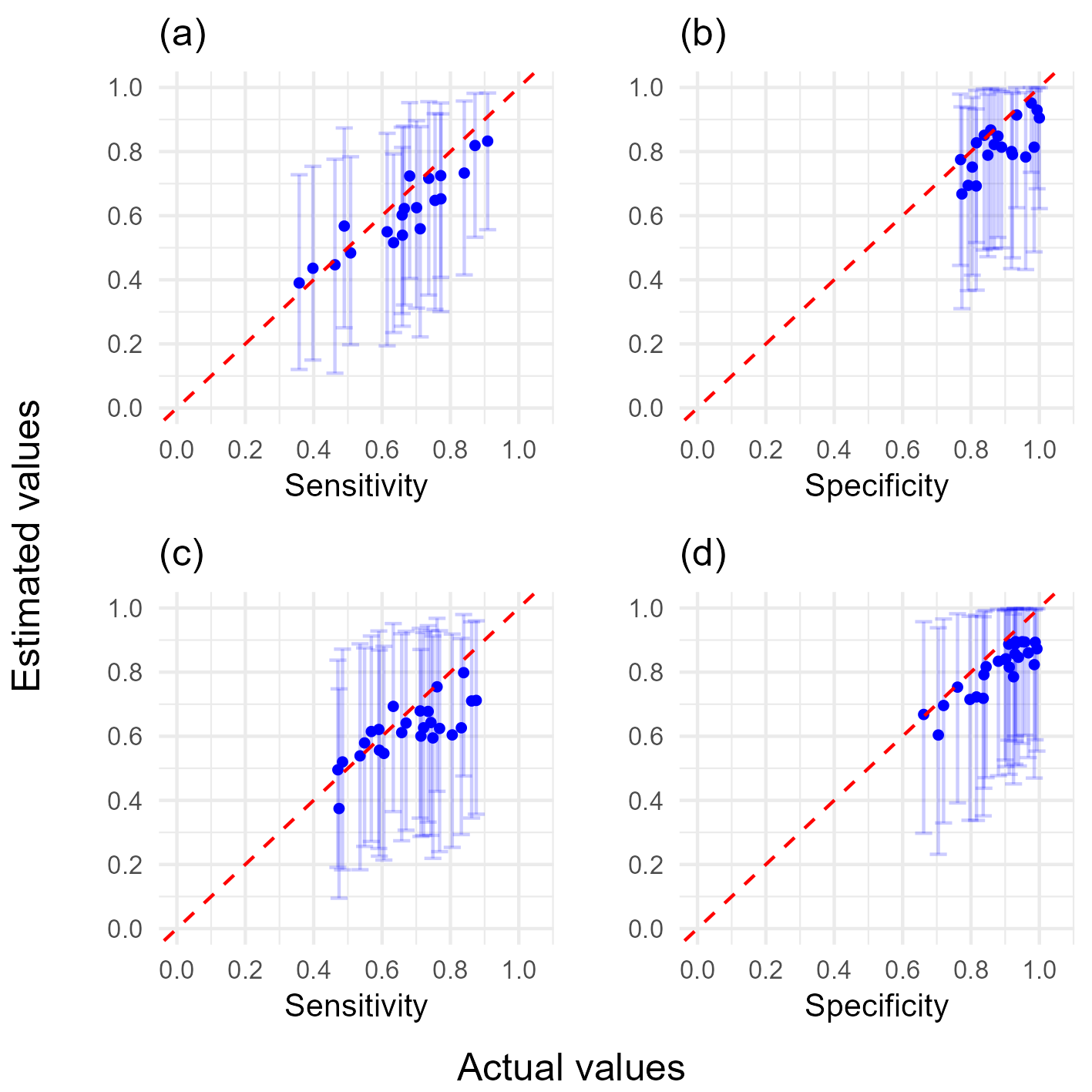

Supplement: S7 Fig — Poor sensitivity and specificity prior distributions were used with reliable prior knowledge of true disease prevalence with (a, b) no covariance model with no simulated covariance, (c, d) covariance model with simulated covariance. In both instances 100 trees each were assessed in higher and lower true disease prevalence locations. Error bars represent 95% confidence intervals, and dashed line represents perfect agreement between estimated values and actual values. (TIF) [file pcbi.1012957.s011.tif]

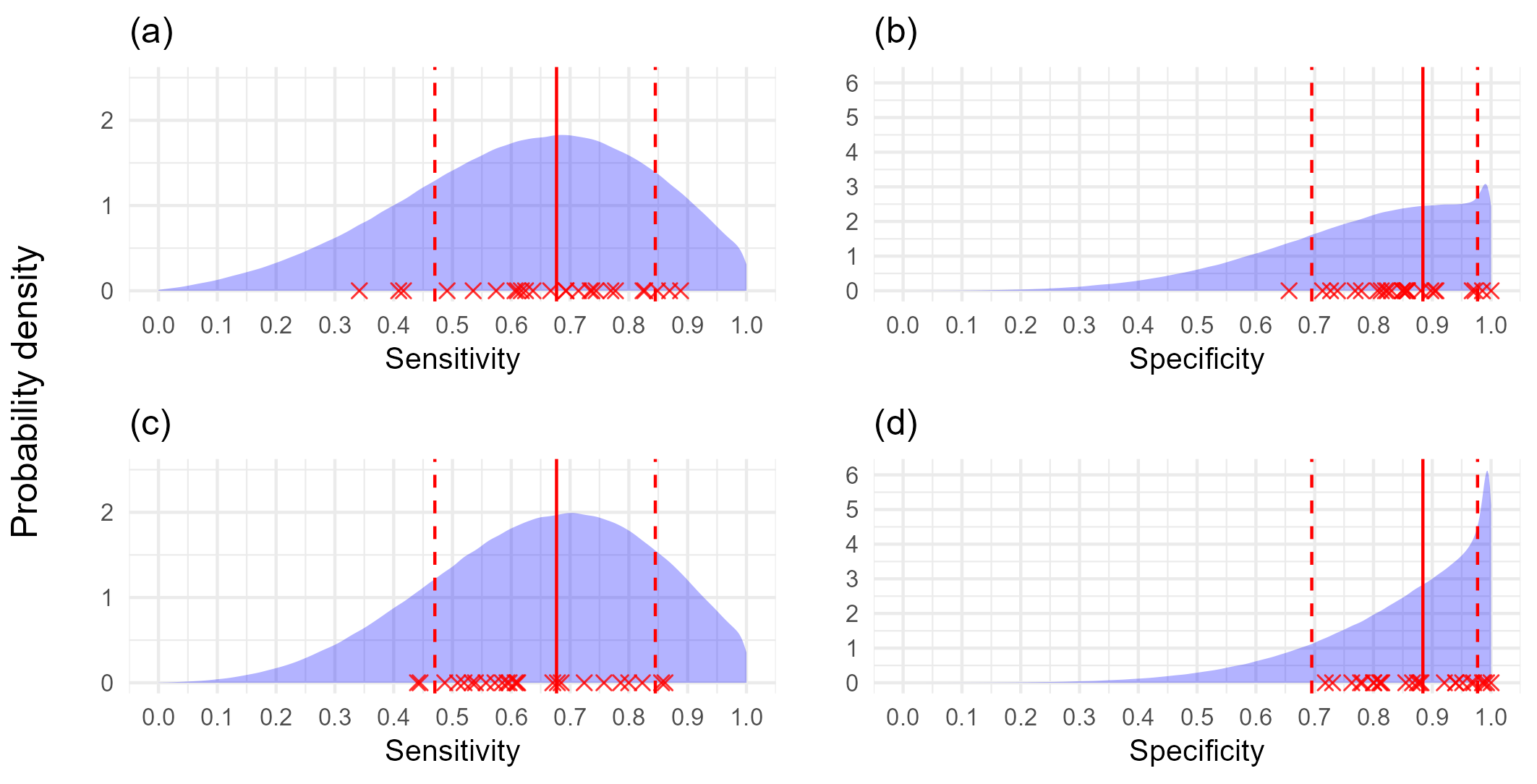

Supplement: S8 Fig — Probability density of estimated sensitivity and specificity of surveyors using poor sensitivity and specificity prior distributions for symptom one, and very good sensitivity and specificity prior distributions for symptom two with reliable prior knowledge of true disease prevalence with (a, b) no covariance model with no simulated covariance, (c, d) covariance model with simulated covariance. In both instances 100 trees each were assessed in higher and lower true disease prevalence locations by 25 surveyors. Red crosses represent true surveyor sensitivity and specificity. Solid red line represents the 50th percentile (median), dotted red lines represent the 5th and 95th percentiles of the distributions surveyor sensitivity and specificity values were generated from. (TIF) [file pcbi.1012957.s012.tif]

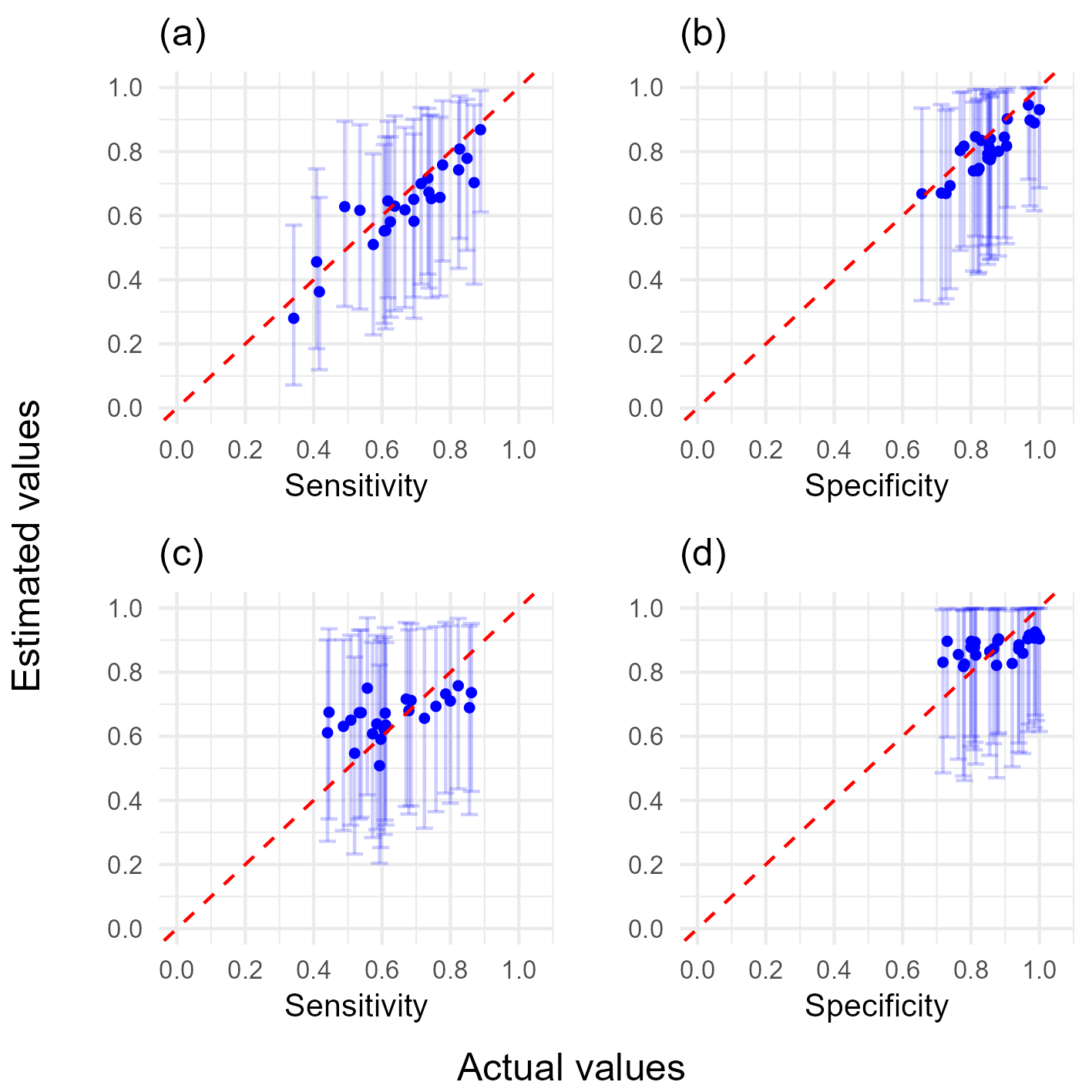

Supplement: S9 Fig — Poor sensitivity and specificity prior distributions for symptom one, and very good sensitivity and specificity prior distributions for symptom two were used with reliable prior knowledge of true disease prevalence with (a, b) no covariance model with no simulated covariance, (c, d) covariance model with simulated covariance. In both instances 100 trees each were assessed in higher and lower true disease prevalence locations. Error bars represent 95% confidence intervals, and dashed line represents perfect agreement between estimated values and actual values. (TIF) [file pcbi.1012957.s013.tif]

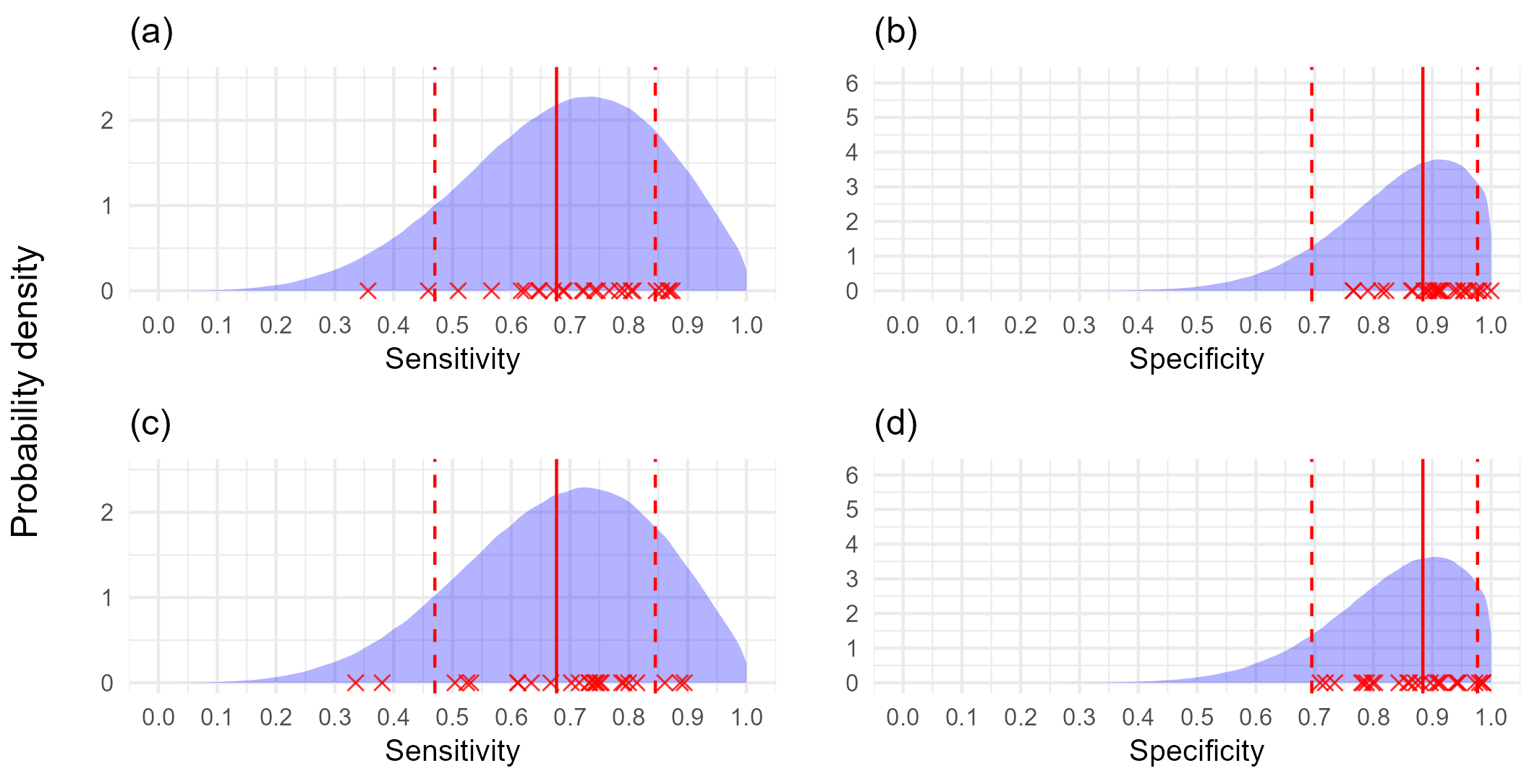

Supplement: S10 Fig — Probability density of estimated sensitivity and specificity of surveyors using good sensitivity and specificity prior distributions with reliable prior knowledge of true disease prevalence with (a, b) no covariance model with no simulated covariance, (c, d) covariance model with simulated covariance. In both instances 100 trees each were assessed in higher and lower true disease prevalence locations by 25 surveyors. Red crosses represent true surveyor sensitivity and specificity. Solid red line represents the 50th percentile (median), dotted red lines represent the 5th and 95th percentiles of the distributions surveyor sensitivity and specificity values were generated from. (TIF) [file pcbi.1012957.s014.tif]

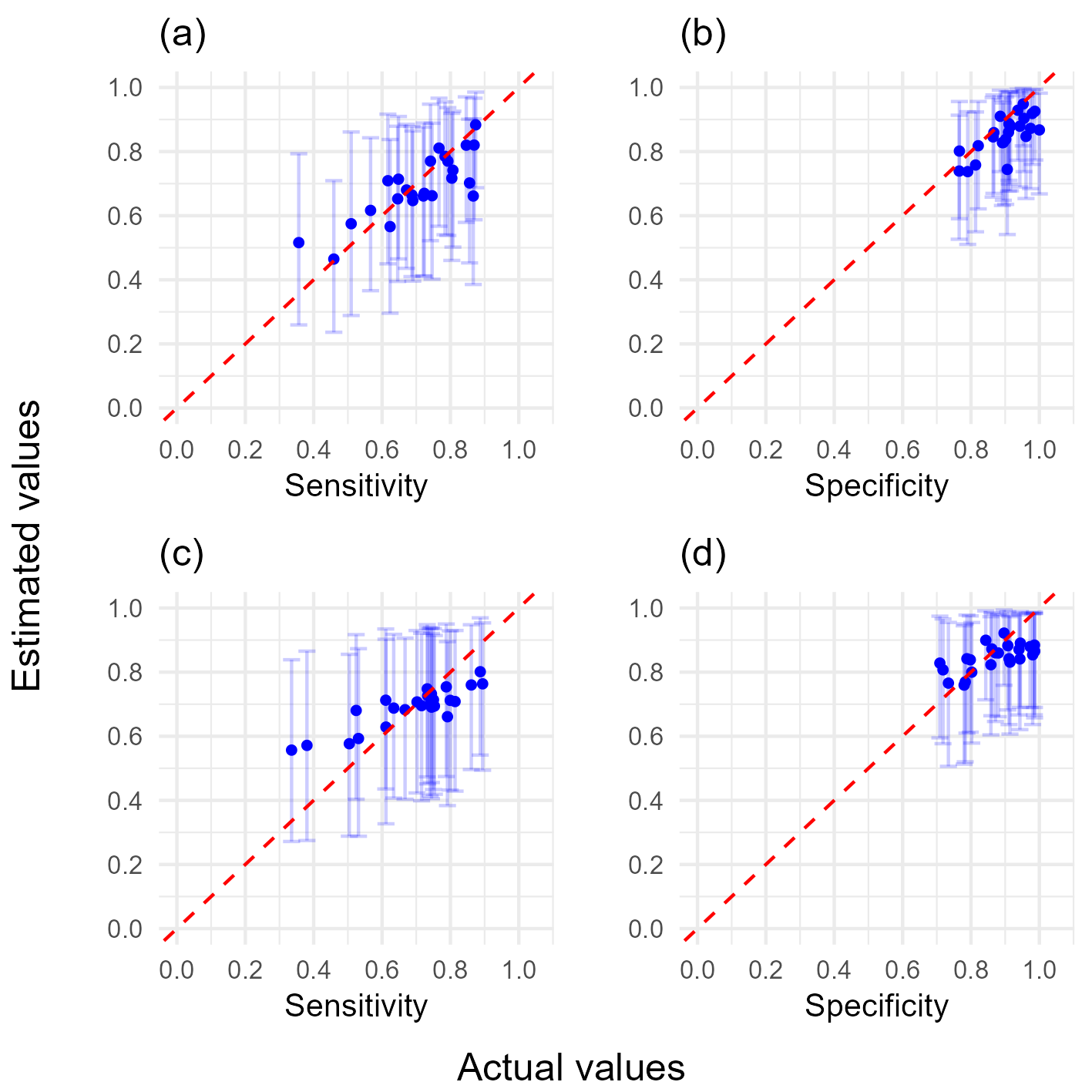

Supplement: S11 Fig — Good sensitivity and specificity prior distributions were used with reliable prior knowledge of true disease prevalence with (a, b) no covariance model with no simulated covariance, (c, d) covariance model with simulated covariance. In both instances 100 trees each were assessed in higher and lower true disease prevalence locations. Error bars represent 95% confidence intervals, and dashed line represents perfect agreement between estimated values and actual values. (TIF) [file pcbi.1012957.s015.tif]

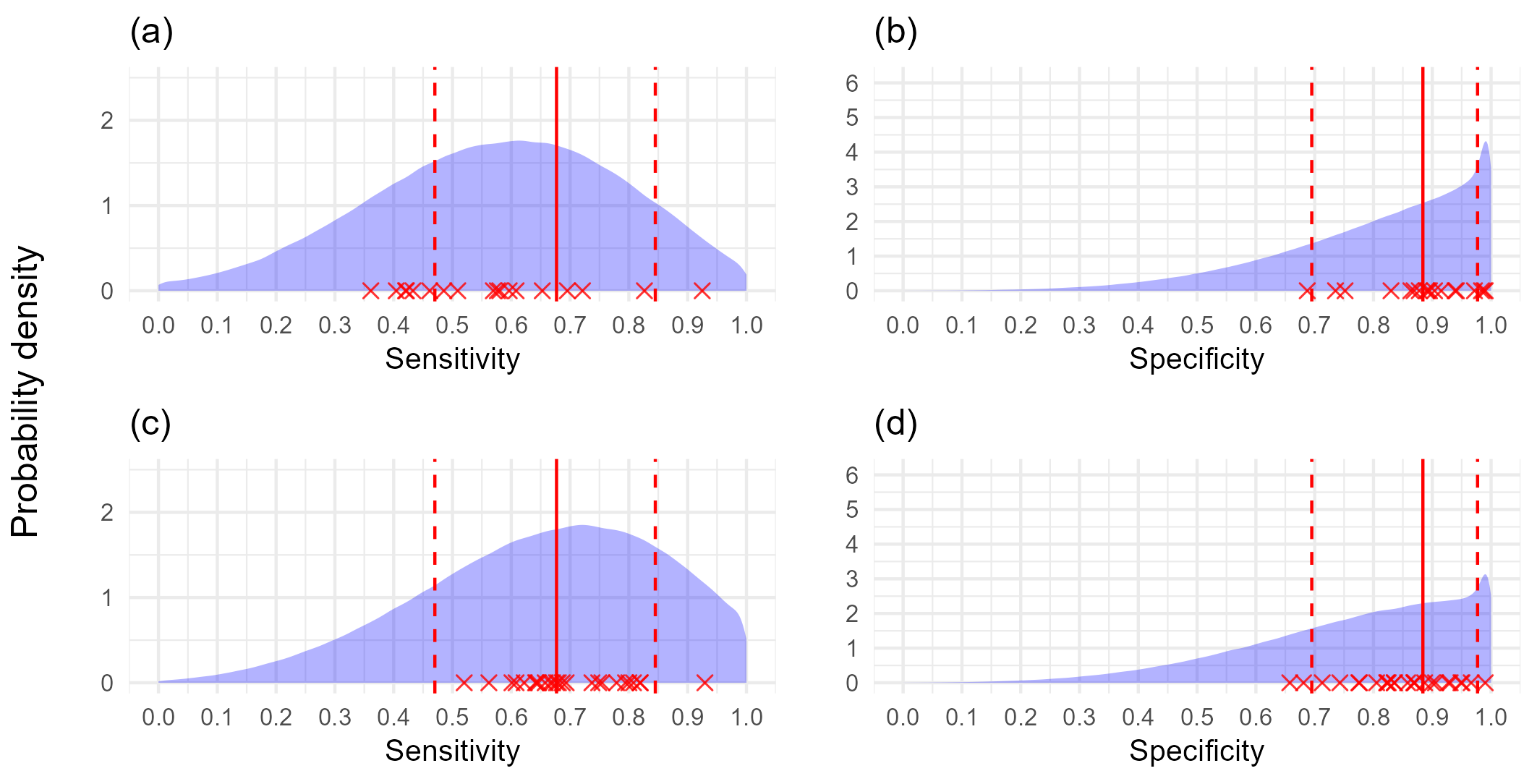

Supplement: S12 Fig — Probability density of estimated sensitivity and specificity of surveyors using poor sensitivity and specificity prior distributions with reliable prior knowledge of true disease prevalence with (a, b) no covariance model with no simulated covariance, (c, d) covariance model with simulated covariance. In both instances 120 trees each were assessed in higher and lower true disease prevalence locations by 25 surveyors, but 7/25 models did not adequately converge for the no covariance model and were discarded. Red crosses represent true surveyor sensitivity and specificity. Solid red line represents the 50th percentile (median), dotted red lines represent the 5th and 95th percentiles of the distributions surveyor sensitivity and specificity values were generated from. (TIF) [file pcbi.1012957.s016.tif]

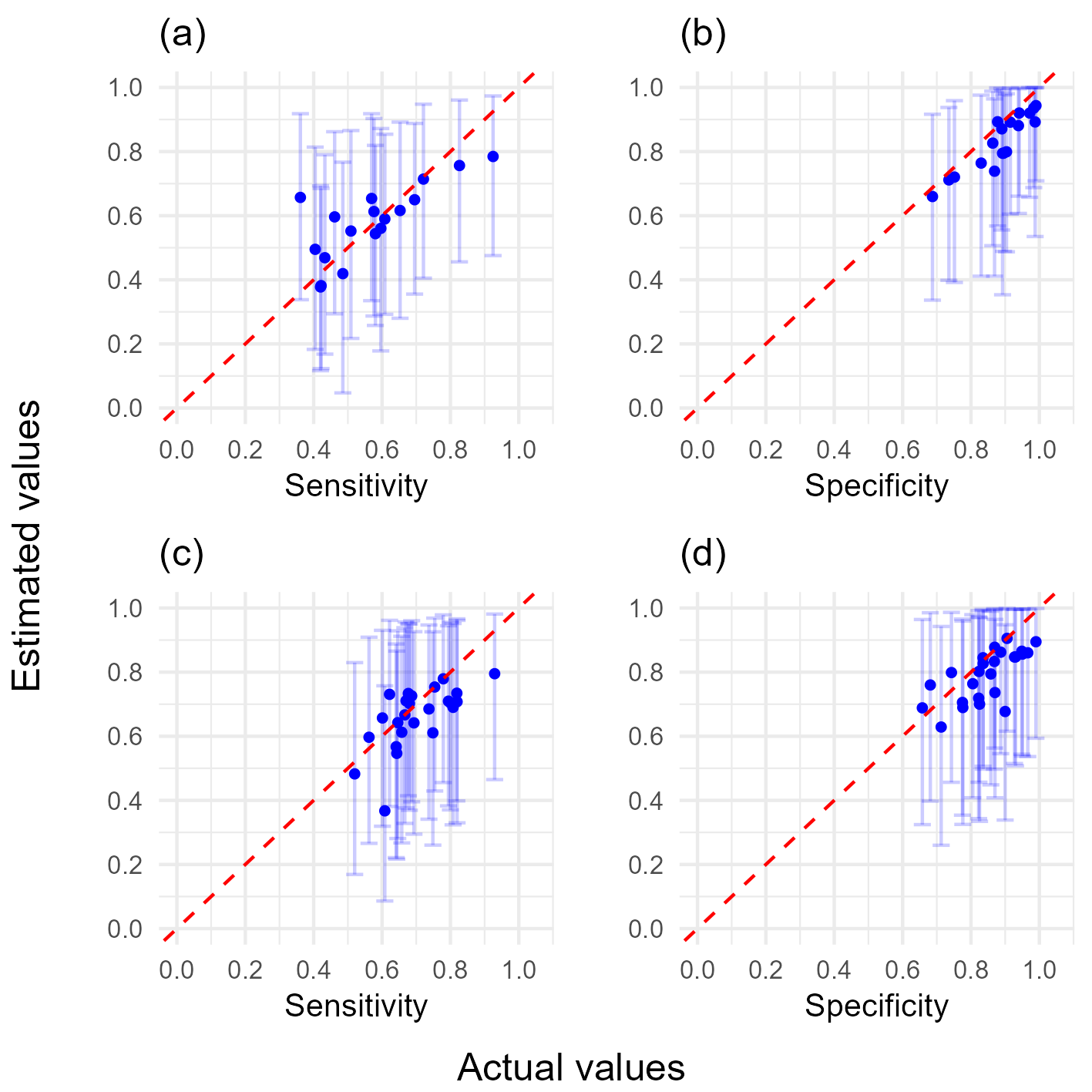

Supplement: S13 Fig — Poor sensitivity and specificity prior distributions with reliable prior knowledge of true disease prevalence with (a, b) no covariance model with no simulated covariance, (c, d) covariance model with simulated covariance. In both instances 120 trees each were assessed in higher and lower true disease prevalence locations. Error bars represent 95% confidence intervals, and dashed line represents perfect agreement between estimated values and actual values. (TIF) [file pcbi.1012957.s017.tif]

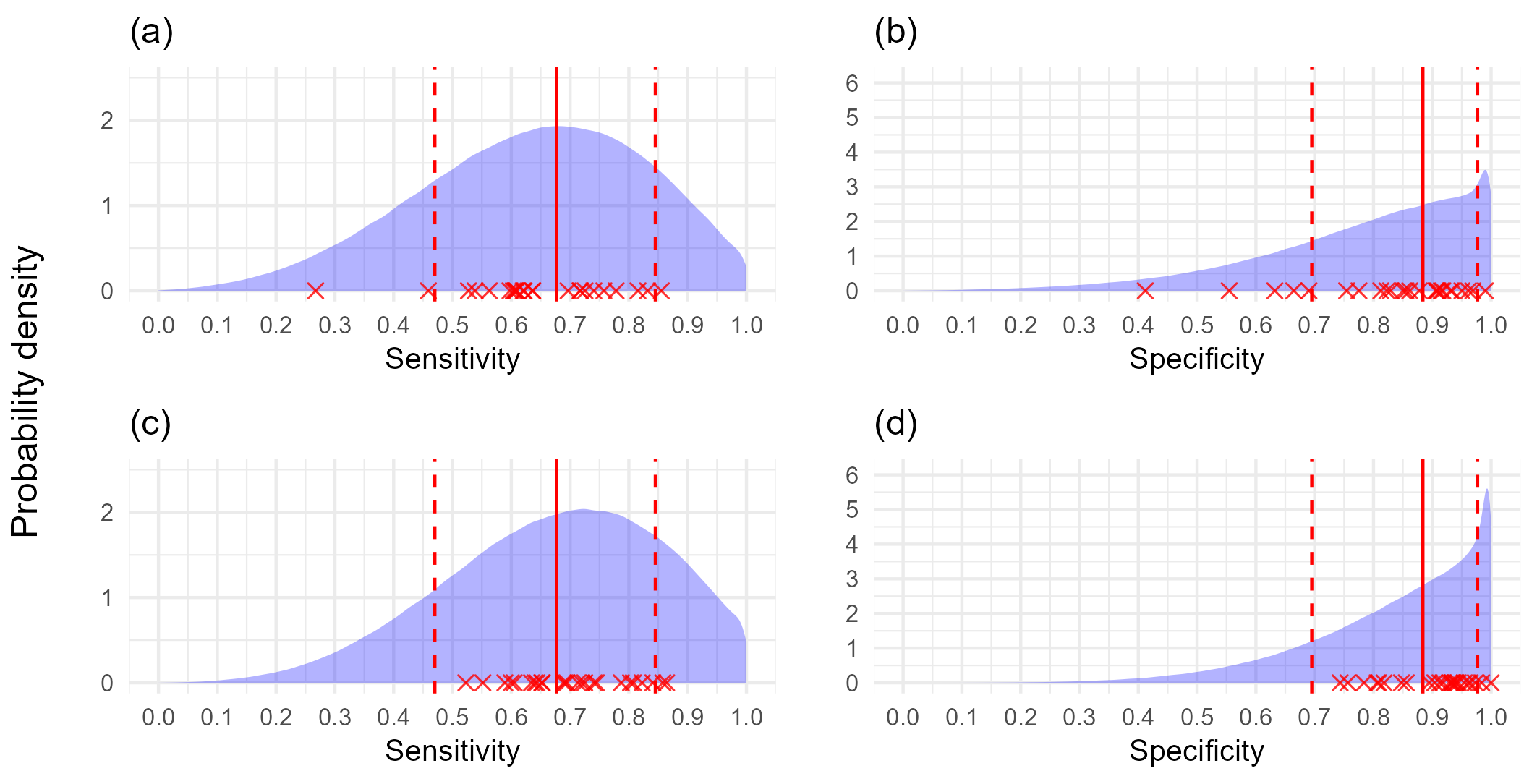

Supplement: S14 Fig — Probability density of estimated sensitivity and specificity of surveyors using poor sensitivity and specificity prior distributions for symptom one, and very good sensitivity and specificity prior distributions for symptom two with reliable prior knowledge of true disease prevalence with (a, b) no covariance model with no simulated covariance, (c, d) covariance model with simulated covariance. In both instances 120 trees each were assessed in higher and lower true disease prevalence locations by 25 surveyors. Red crosses represent true surveyor sensitivity and specificity. Solid red line represents the 50th percentile (median), dotted red lines represent the 5th and 95th percentiles of the distributions surveyor sensitivity and specificity values were generated from. (TIF) [file pcbi.1012957.s018.tif]

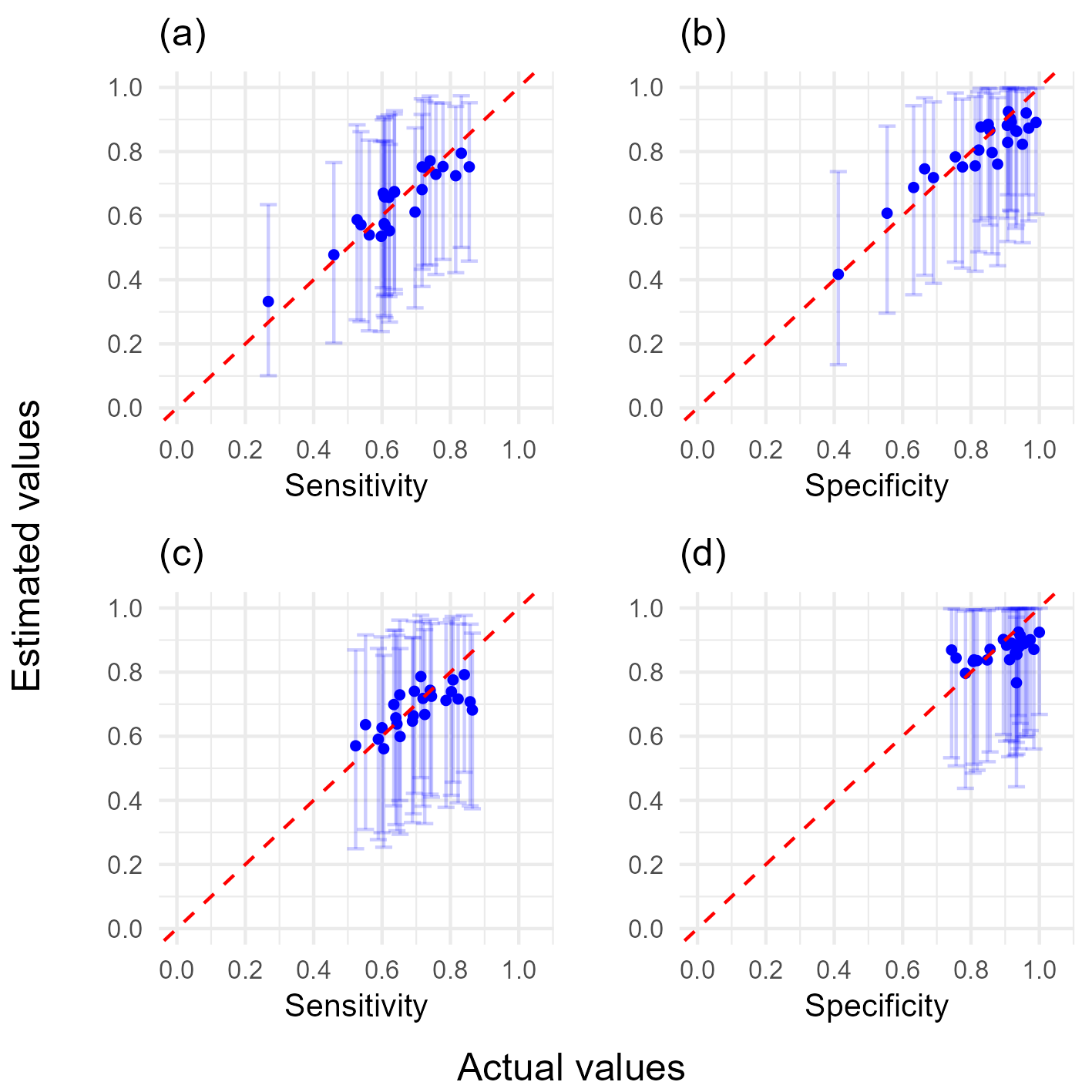

Supplement: S15 Fig — Poor sensitivity and specificity prior distributions for symptom one, and very good sensitivity and specificity prior distributions for symptom two were used with reliable prior knowledge of true disease prevalence with (a, b) no covariance model with no simulated covariance, (c, d) covariance model with simulated covariance. In both instances 120 trees each were assessed in higher and lower true disease prevalence locations. Error bars represent 95% confidence intervals, and dashed line represents perfect agreement between estimated values and actual values. (TIF) [file pcbi.1012957.s019.tif]

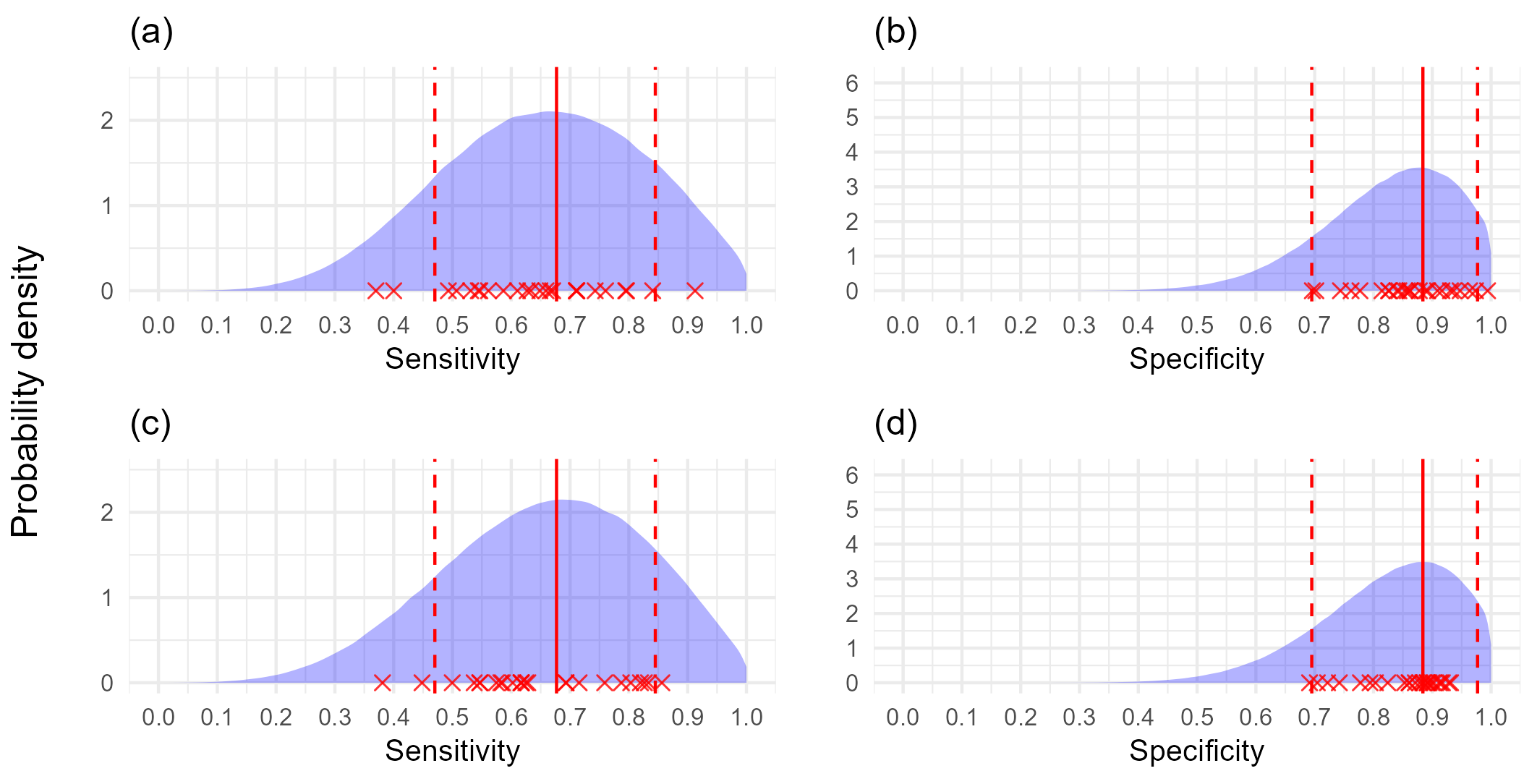

Supplement: S16 Fig — Probability density of estimated sensitivity and specificity of surveyors using good sensitivity and specificity prior distributions with reliable prior knowledge of true disease prevalence with (a, b) no covariance model with no simulated covariance, (c, d) covariance model with simulated covariance. In both instances 120 trees each were assessed in higher and lower true disease prevalence locations by 25 surveyors. Red crosses represent true surveyor sensitivity and specificity. Solid red line represents the 50th percentile (median), dotted red lines represent the 5th and 95th percentiles of the distributions surveyor sensitivity and specificity values were generated from. (TIF) [file pcbi.1012957.s020.tif]

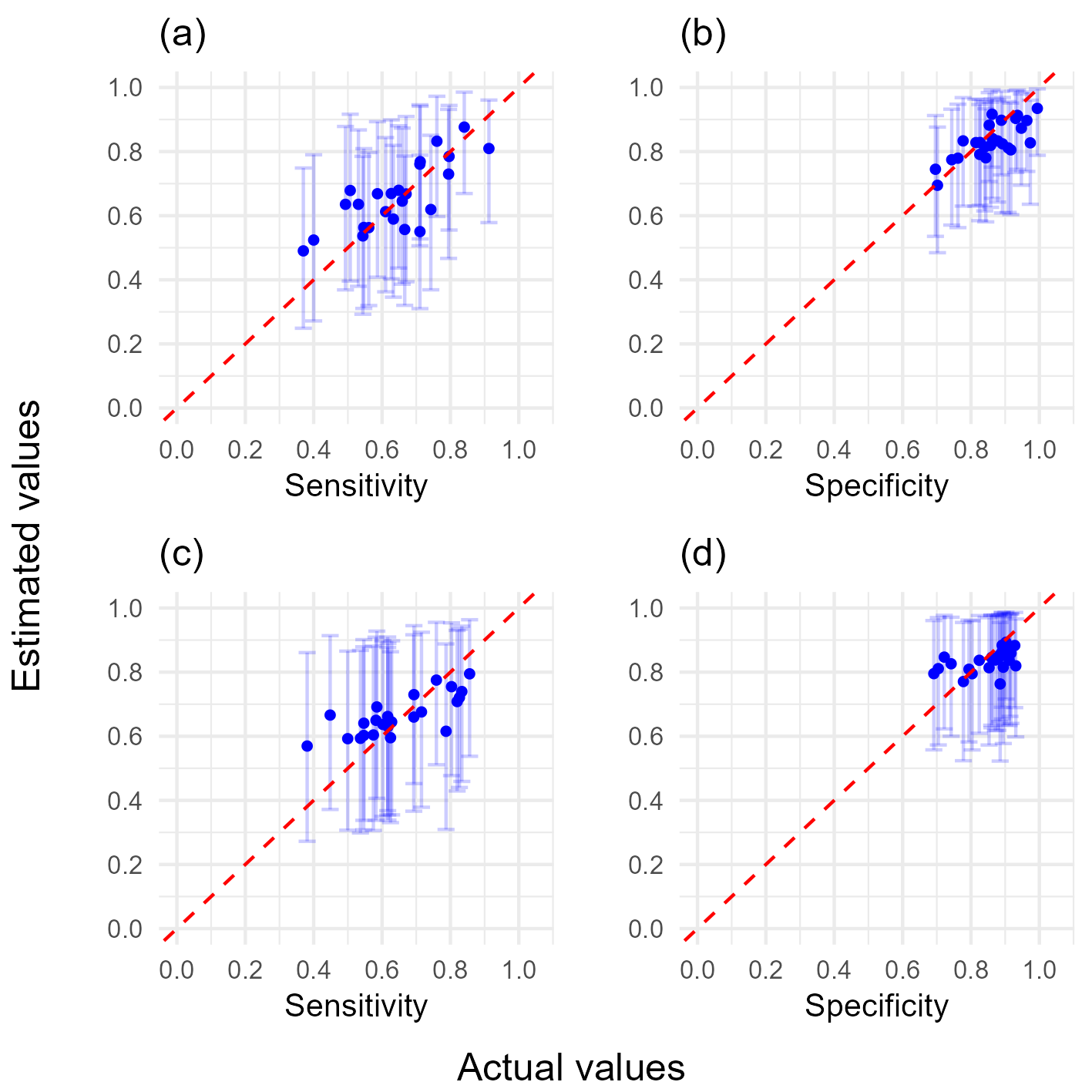

Supplement: S17 Fig — Good sensitivity and specificity prior distributions were used with reliable prior knowledge of true disease prevalence with (a, b) no covariance model with no simulated covariance, (c, d) covariance model with simulated covariance. In both instances 120 trees each were assessed in higher and lower true disease prevalence locations. Error bars represent 95% confidence intervals, and dashed line represents perfect agreement between estimated values and actual values. (TIF) [file pcbi.1012957.s021.tif]

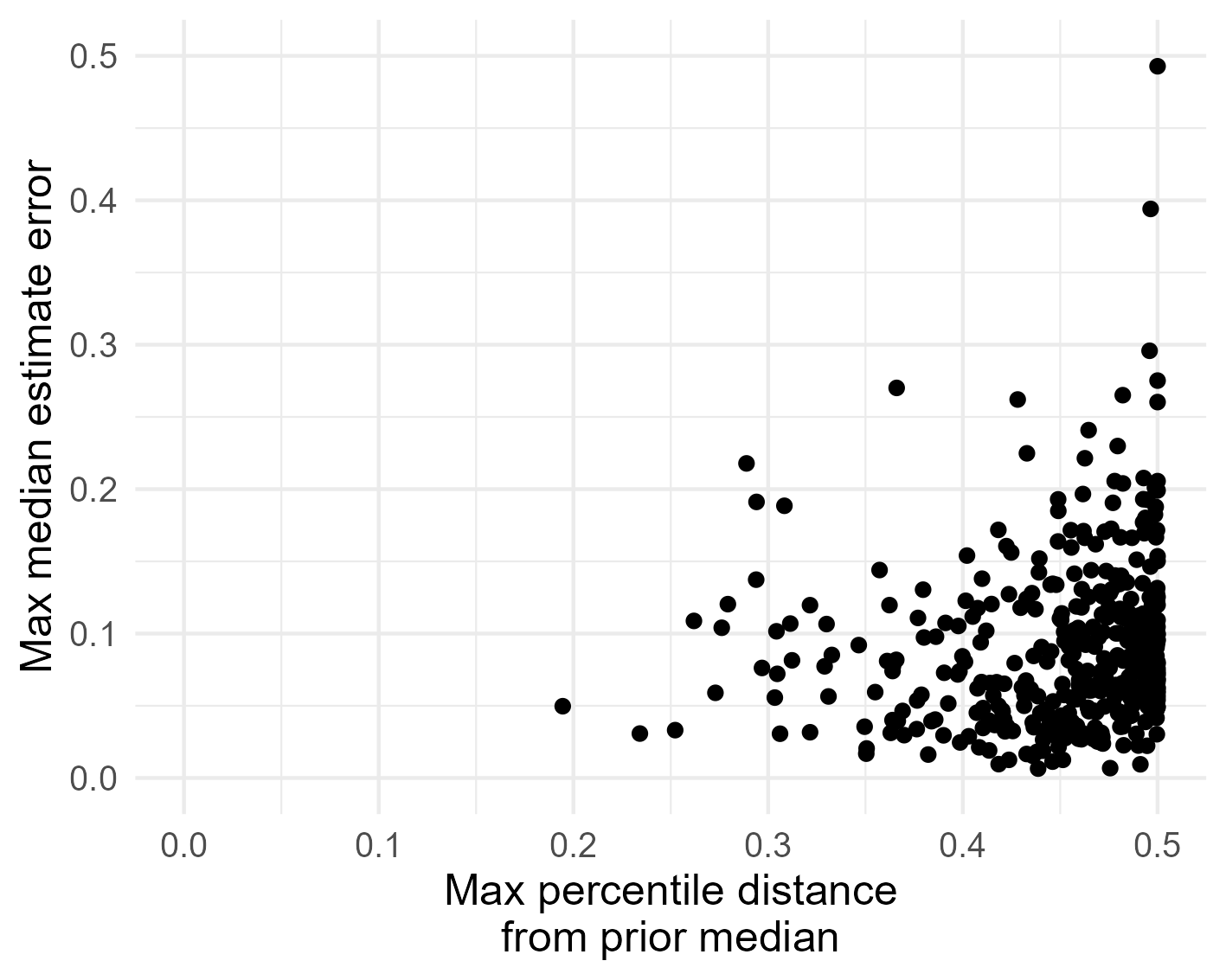

Supplement: S18 Fig — The maximum error (i.e., difference from the actual value of a simulated surveyor) of a median value estimate for either the sensitivity or specificity against the maximum percentile distance of an actual value of a simulated surveyor from the median of a prior distribution (sensitivity, specificity, site true disease prevalences, or covariance parameters). (TIF) [file pcbi.1012957.s022.tif]

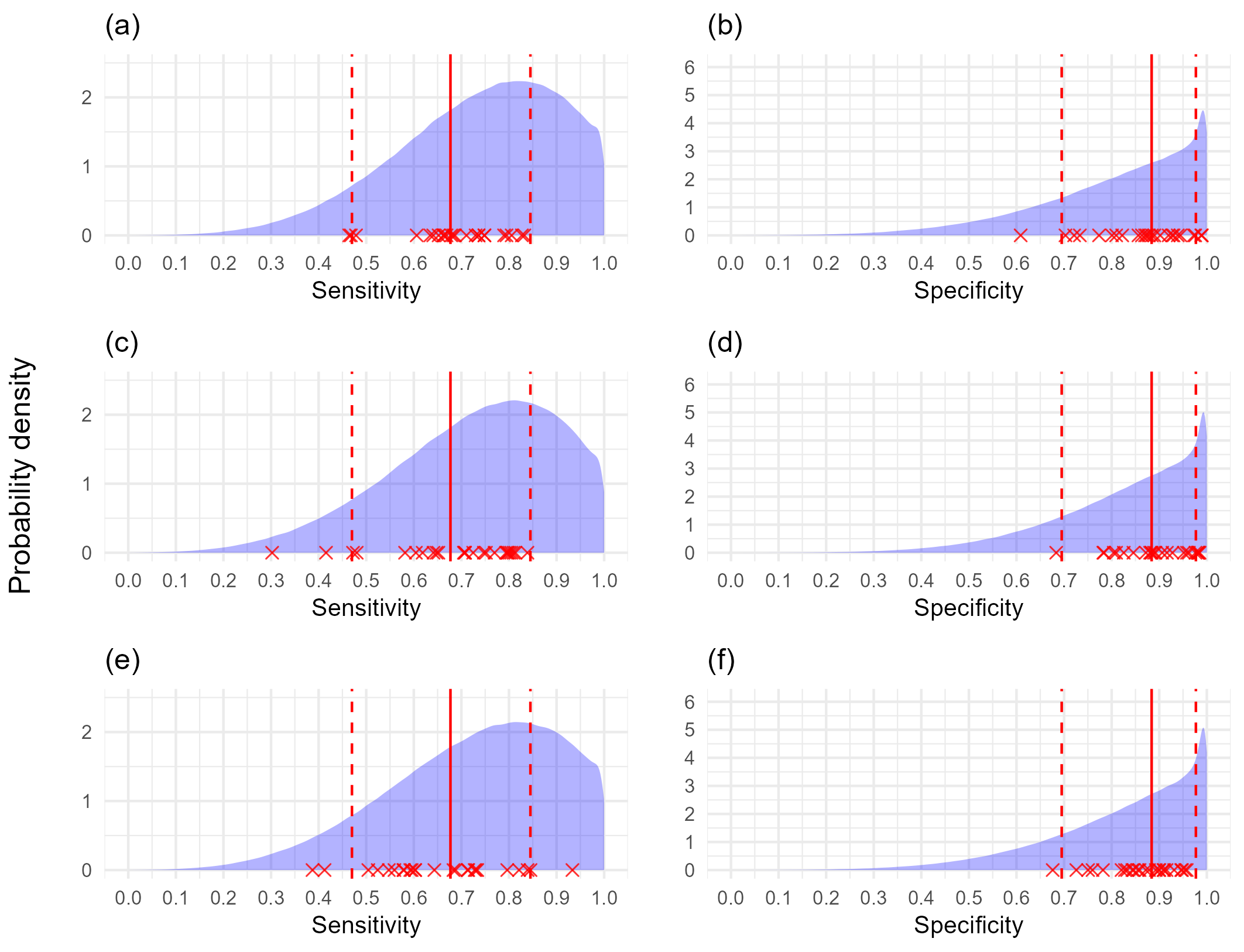

Supplement: S19 Fig — Probability density of estimated sensitivity and specificity of surveyors using poor sensitivity and specificity prior distributions for symptom one, and very good sensitivity and specificity prior distributions for symptom two with misspecified prior knowledge which underestimated the true disease prevalence at survey locations, with 25 surveyors each assessing (a, b) 80, (c, d) 100, or (e, f) 120 trees in each of the higher and lower true disease prevalence locations. Red crosses represent true surveyor sensitivity and specificity. Solid red line represents the 50th percentile (median), dotted red lines represent the 5th and 95th percentiles of the distributions surveyor sensitivity and specificity values were generated from. (TIF) [file pcbi.1012957.s023.tif]

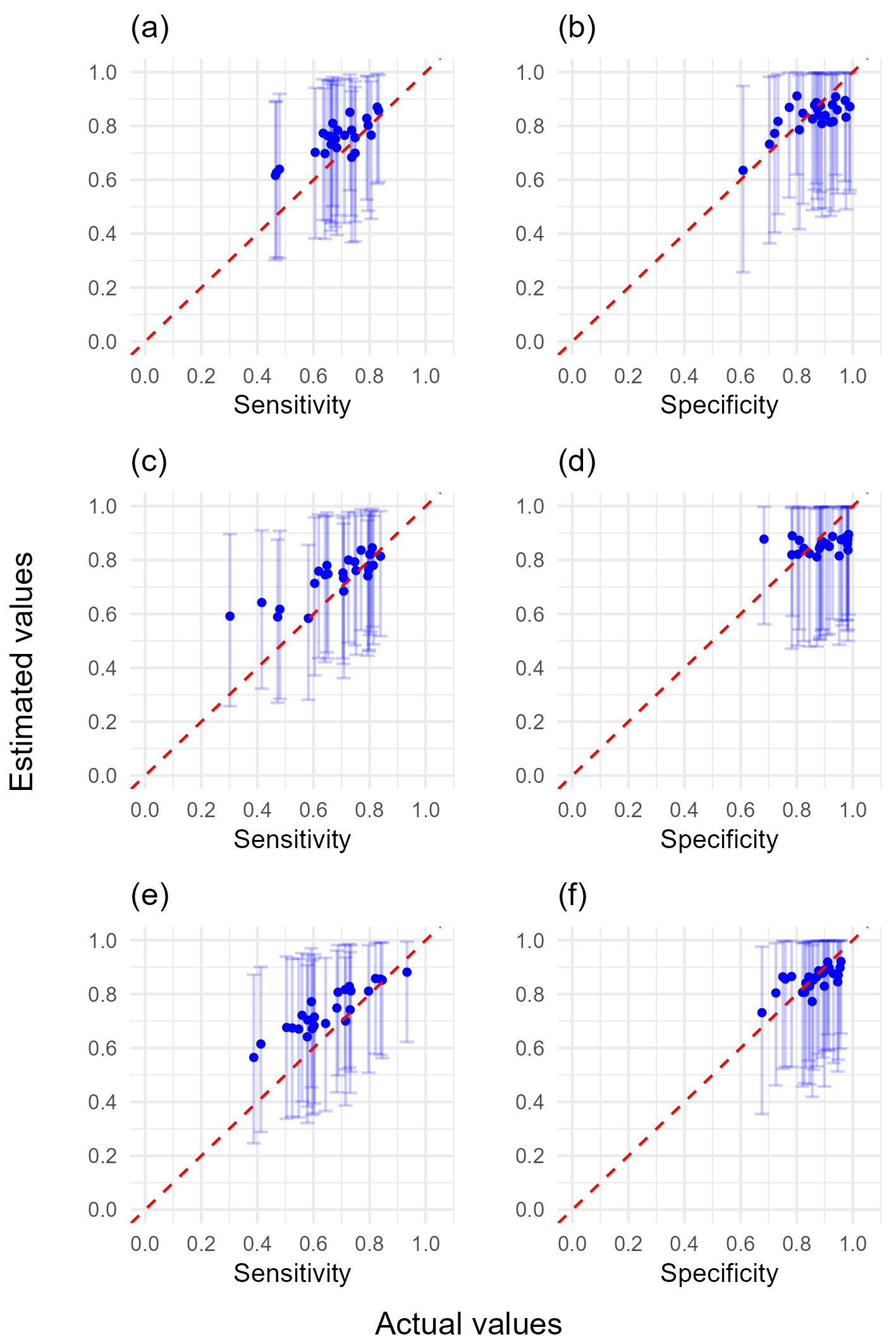

Supplement: S20 Fig — Poor sensitivity and specificity prior distributions for symptom one, and very good sensitivity and specificity prior distributions for symptom two were used with misspecified prior knowledge which underestimated the true disease prevalence at survey locations, with surveyors assessing (a, b) 80, (c, d) 100, or (e, f) 120 trees in each of the higher and lower true disease prevalence locations. Error bars represent 95% confidence intervals, and dashed line represents perfect agreement between estimated values and actual values. (TIF) [file pcbi.1012957.s024.tif]

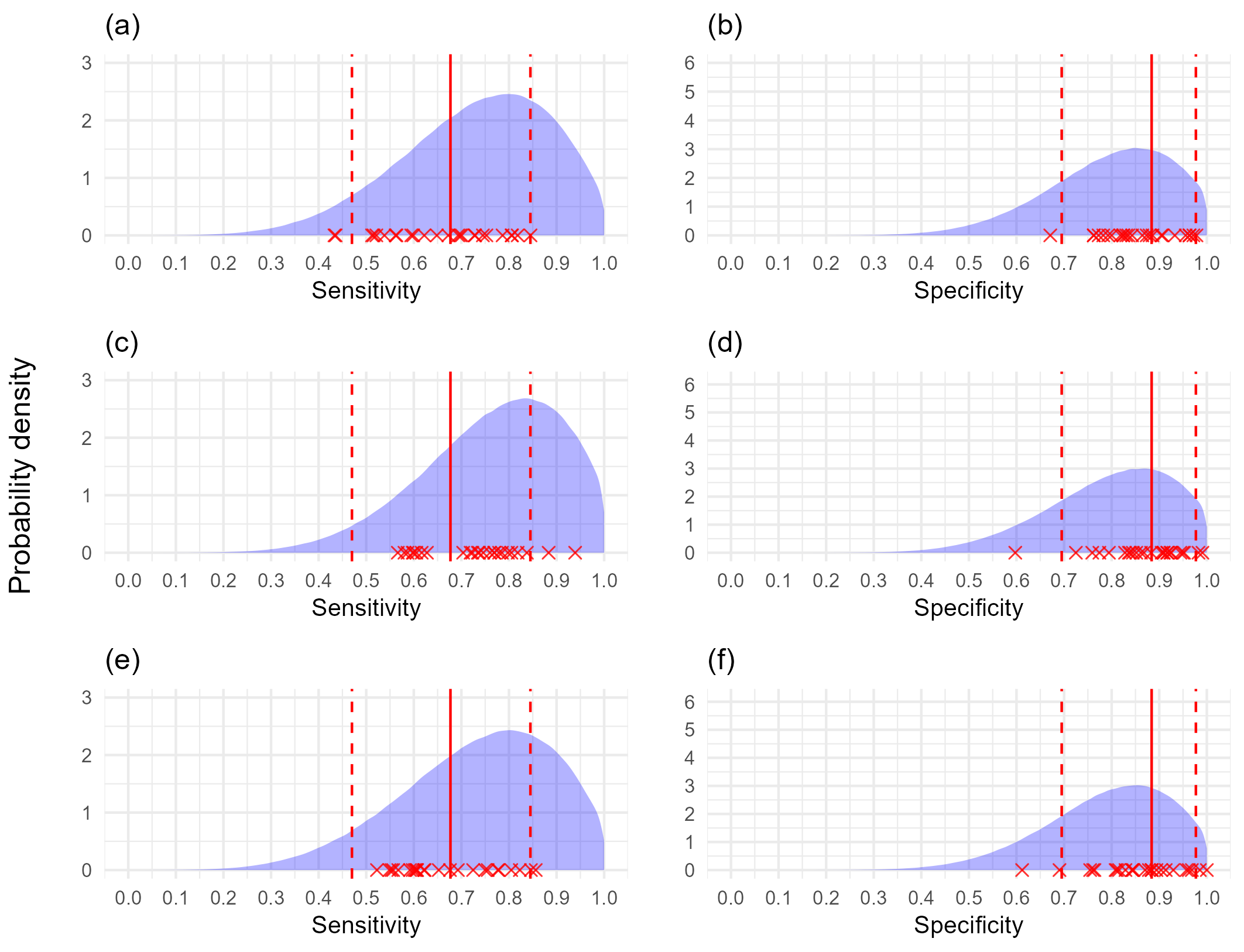

Supplement: S21 Fig — Probability density of estimated sensitivity and specificity of surveyors using good sensitivity and specificity prior distributions with misspecified prior knowledge which underestimated the true disease prevalence at survey locations, with 25 surveyors each assessing (a, b) 80, (c, d) 100, or (e, f) 120 trees in each of the higher and lower true disease prevalence locations. Red crosses represent true surveyor sensitivity and specificity. Solid red line represents the 50th percentile (median), dotted red lines represent the 5th and 95th percentiles of the distributions surveyor sensitivity and specificity values were generated from. (TIF) [file pcbi.1012957.s025.tif]

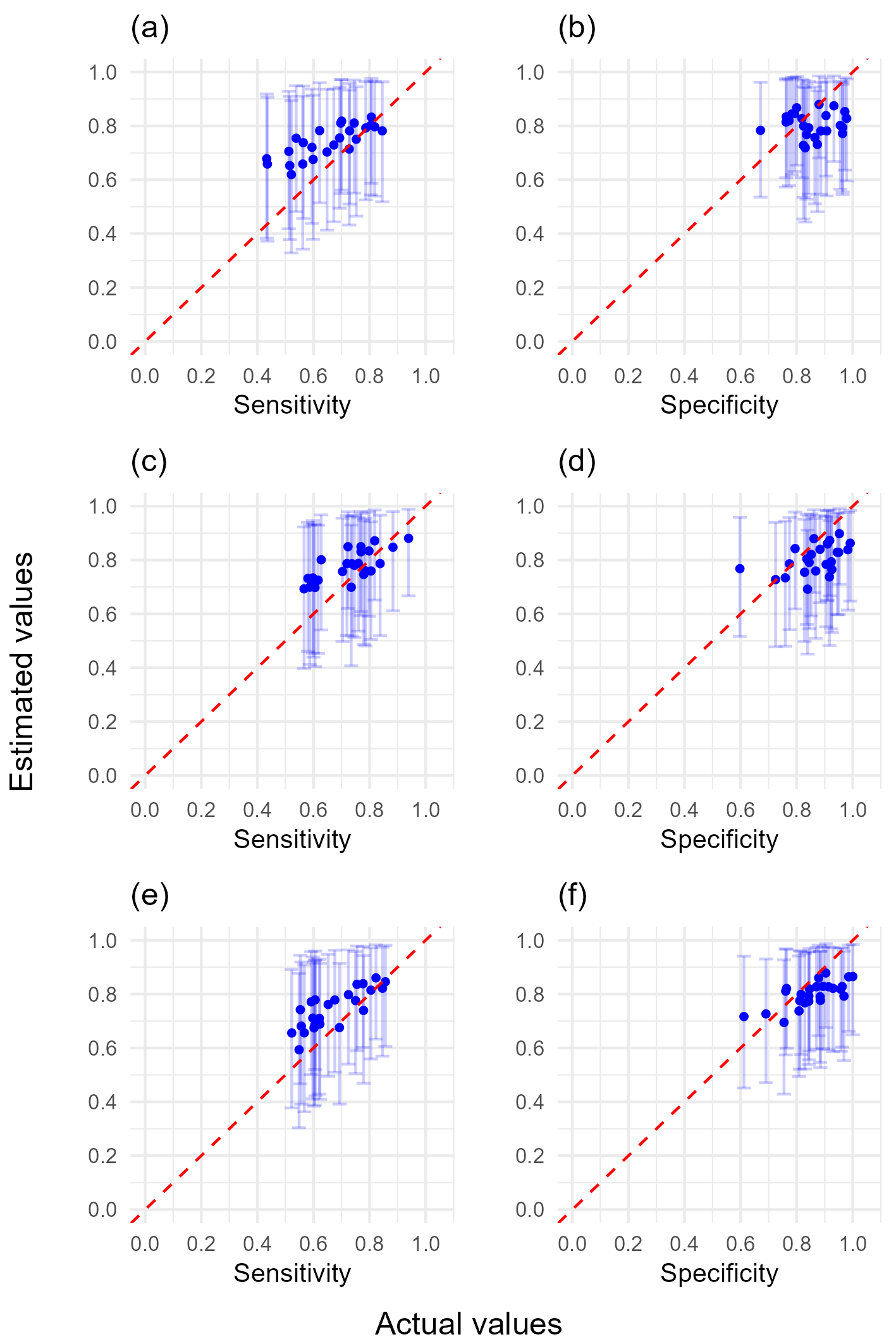

Supplement: S22 Fig — Good sensitivity and specificity prior distributions with misspecified prior knowledge which underestimated the true disease prevalence at survey locations, with surveyors assessing (a, b) 80, (c, d) 100, or (e, f) 120 trees in each of the higher and lower true disease prevalence locations. Error bars represent 95% confidence intervals, and dashed line represents perfect agreement between estimated values and actual values. (TIF) [file pcbi.1012957.s026.tif]

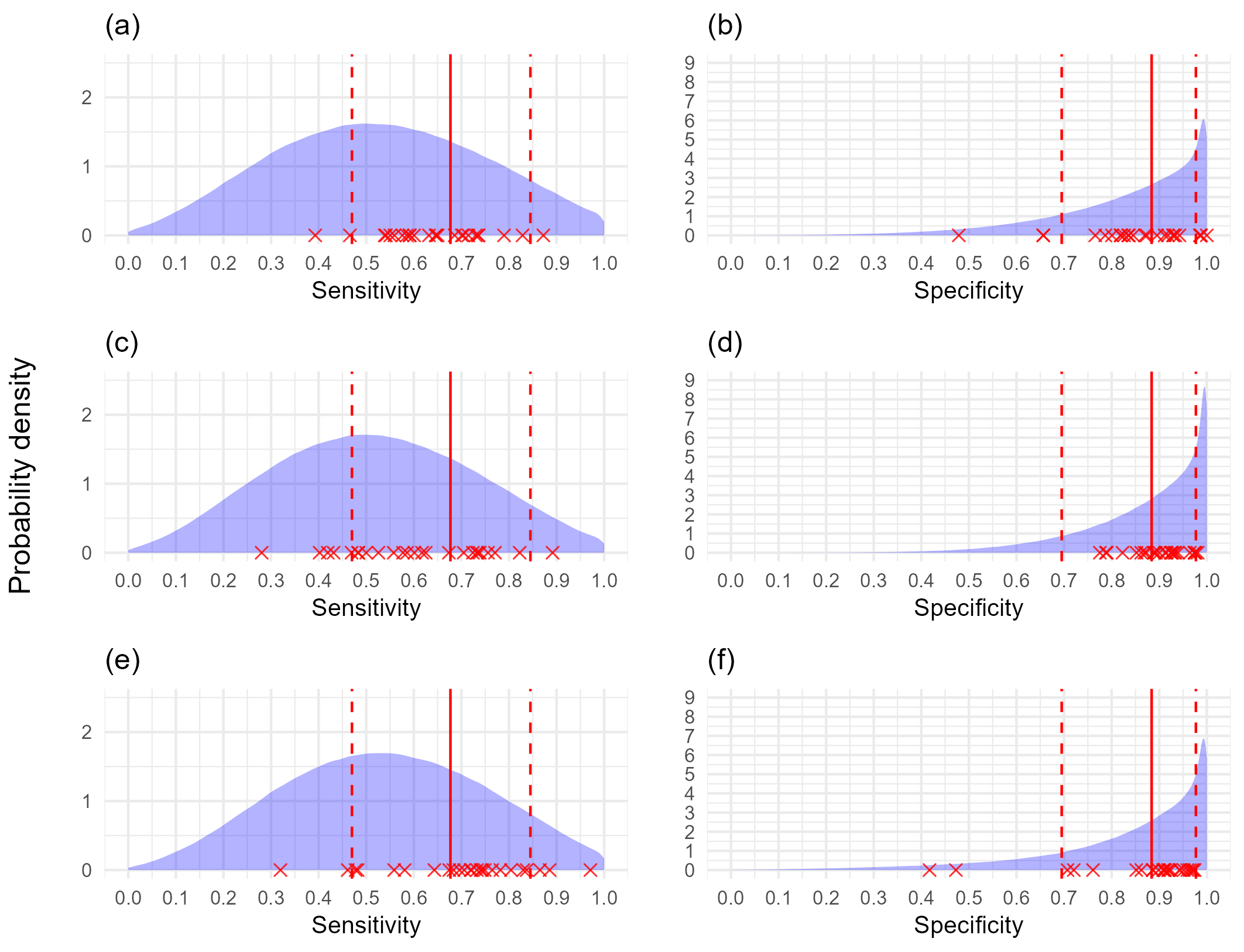

Supplement: S23 Fig — Probability density of estimated sensitivity and specificity of surveyors using poor sensitivity and specificity prior distributions with misspecified prior knowledge which underestimated the true disease prevalence at survey locations, with 25 surveyors each assessing (a, b) 80, (c, d) 100, or (e, f) 120 trees in each of the higher and lower true disease prevalence locations. Red crosses represent true surveyor sensitivity and specificity. Solid red line represents the 50th percentile (median), dotted red lines represent the 5th and 95th percentiles of the distributions surveyor sensitivity and specificity values were generated from. (TIF) [file pcbi.1012957.s027.tif]

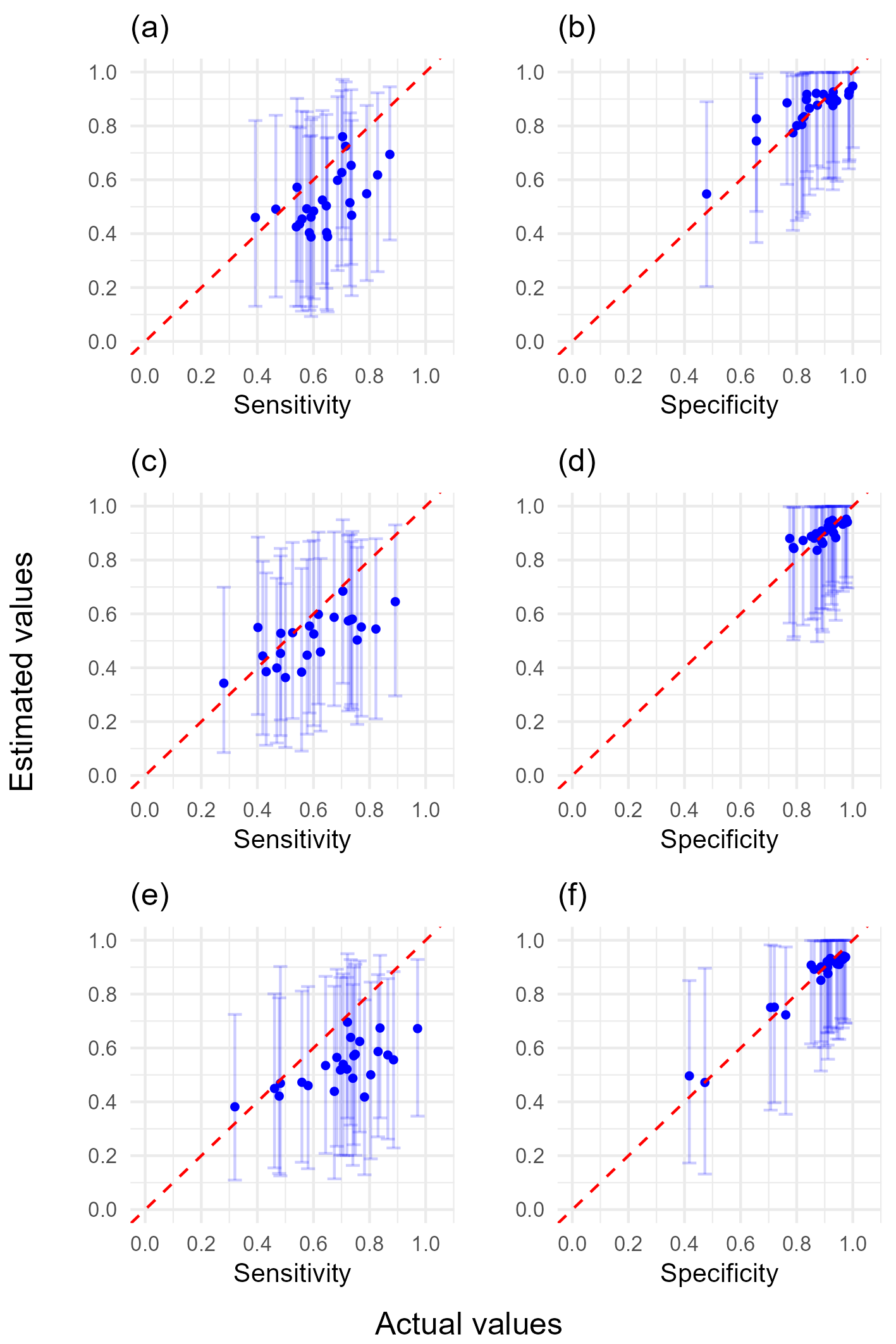

Supplement: S24 Fig — Poor sensitivity and specificity prior distributions were used with misspecified prior knowledge which underestimated the true disease prevalence at survey locations, with surveyors assessing (a, b) 80, (c, d) 100, or (e, f) 120 trees in each of the higher and lower true disease prevalence locations. Error bars represent 95% confidence intervals, and dashed line represents perfect agreement between estimated values and actual values. (TIF) [file pcbi.1012957.s028.tif]

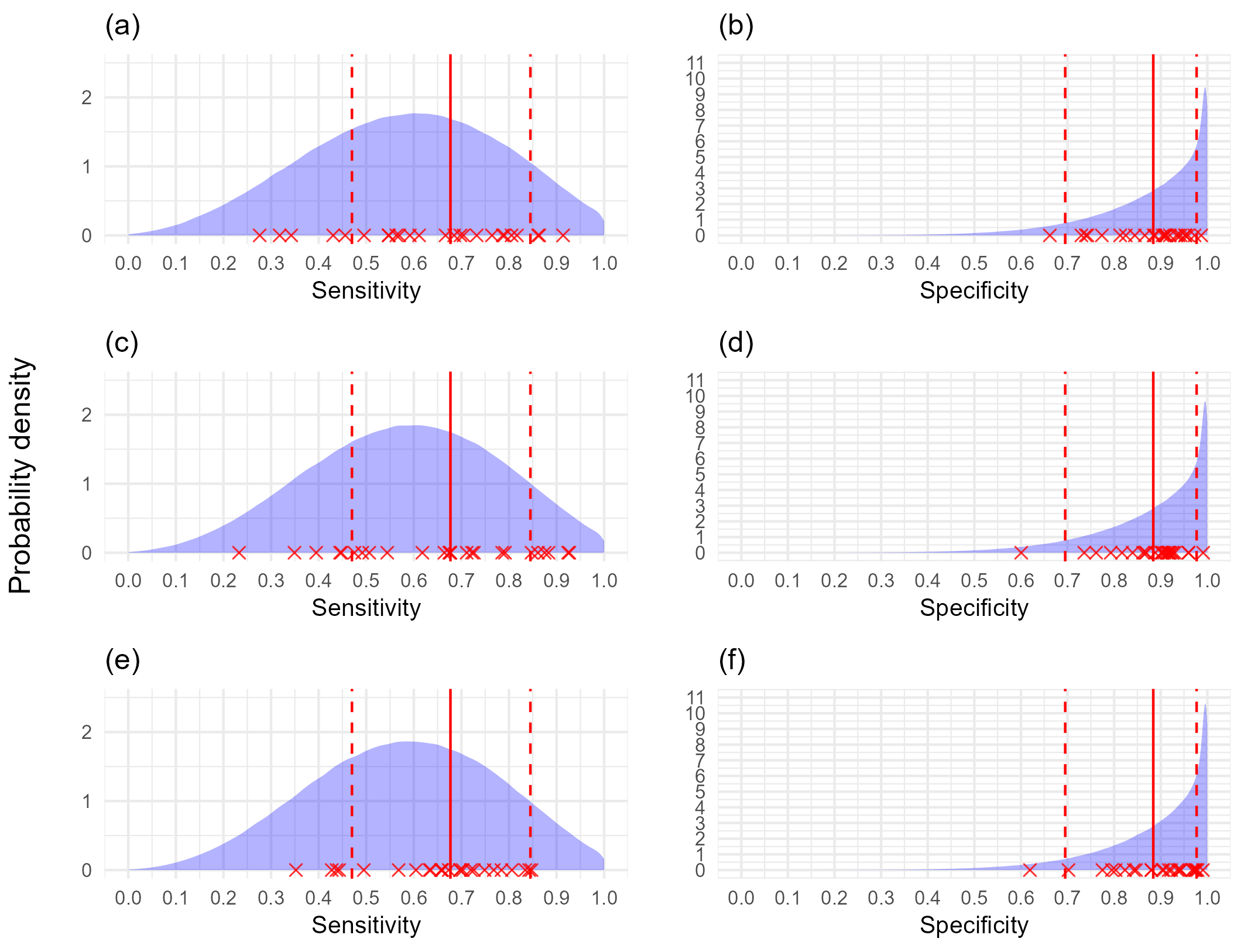

Supplement: S25 Fig — Probability density of estimated sensitivity and specificity of surveyors using poor sensitivity and specificity prior distributions for symptom one, and very good sensitivity and specificity prior distributions for symptom two with misspecified prior knowledge which overestimated the true disease prevalence at survey locations, with 25 surveyors each assessing (a, b) 80, (c, d) 100, or (e, f) 120 trees in each of the higher and lower true disease prevalence locations. Red crosses represent true surveyor sensitivity and specificity. Solid red line represents the 50th percentile (median), dotted red lines represent the 5th and 95th percentiles of the distributions surveyor sensitivity and specificity values were generated from. (TIF) [file pcbi.1012957.s029.tif]

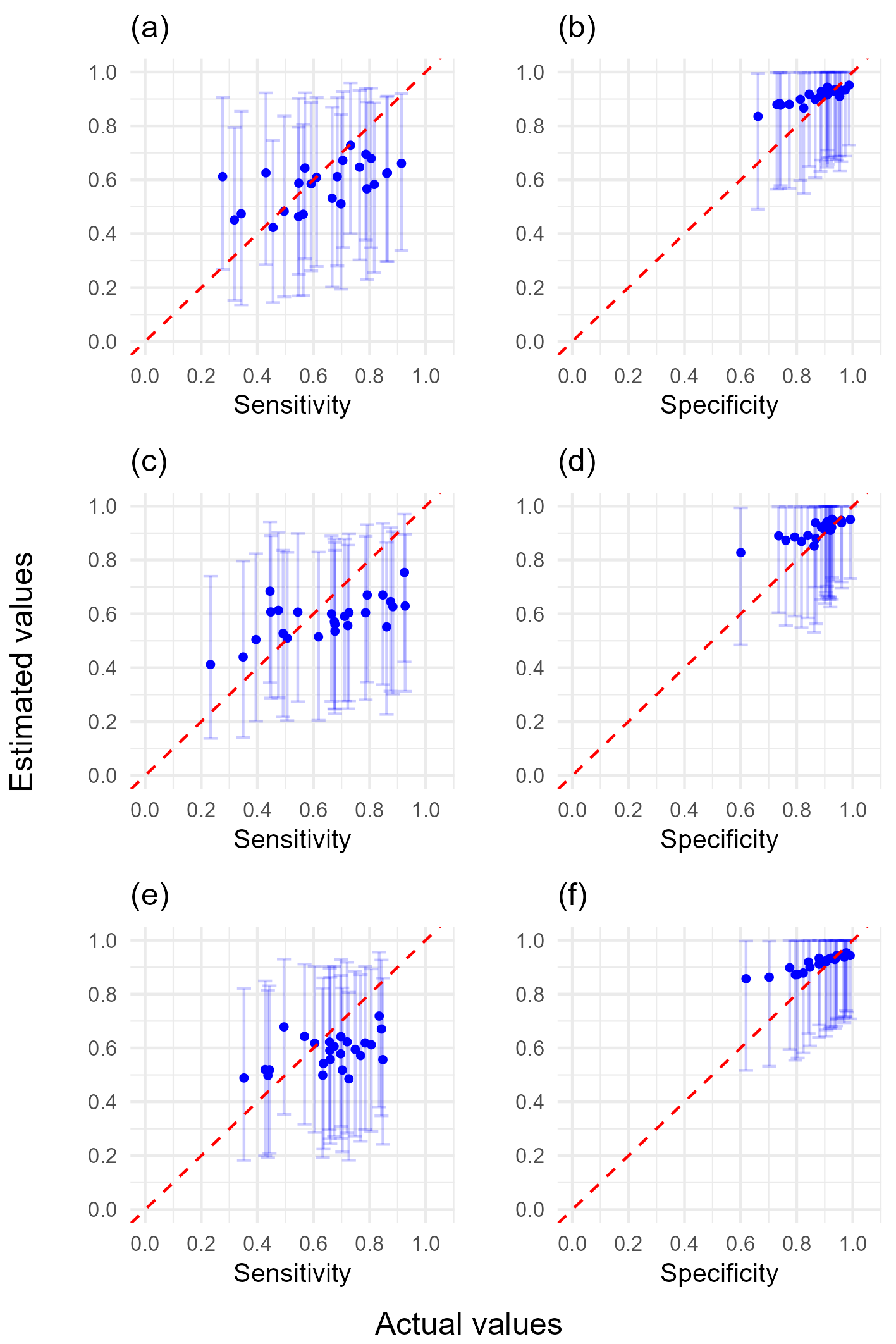

Supplement: S26 Fig — Poor sensitivity and specificity prior distributions for symptom one, and very good sensitivity and specificity prior distributions for symptom two were used with misspecified prior knowledge which overestimated the true disease prevalence at survey locations, with surveyors assessing (a, b) 80, (c, d) 100, or (e, f) 120 trees in each of the higher and lower true disease prevalence locations. Error bars represent 95% confidence intervals, and dashed line represents perfect agreement between estimated values and actual values. (TIF) [file pcbi.1012957.s030.tif]

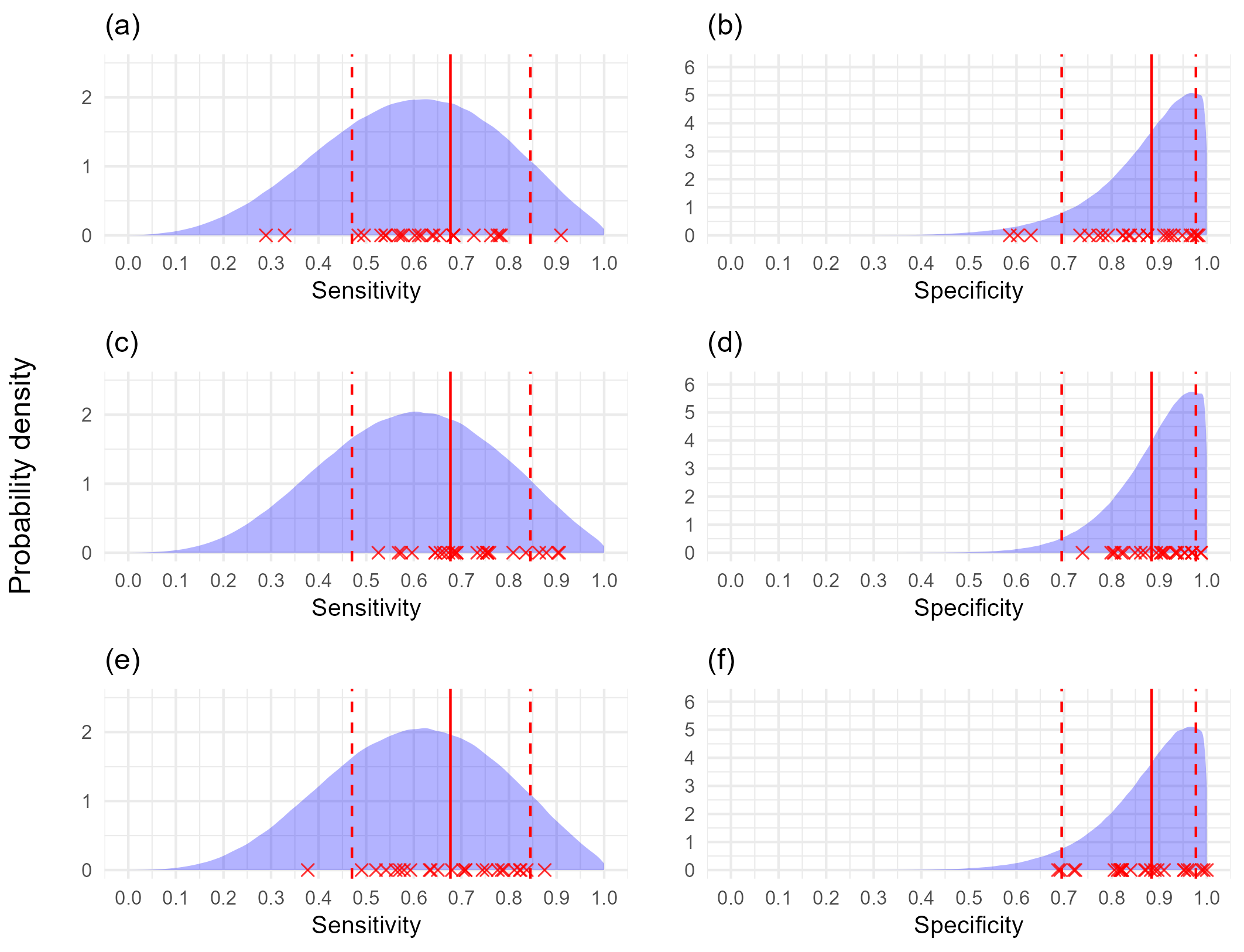

Supplement: S27 Fig — Probability density of estimated sensitivity and specificity of surveyors using good sensitivity and specificity prior distributions with misspecified prior knowledge which overestimated the true disease prevalence at survey locations, with 25 surveyors each assessing (a, b) 80, (c, d) 100, or (e, f) 120 trees in each of the higher and lower true disease prevalence locations. Red crosses represent true surveyor sensitivity and specificity. Solid red line represents the 50th percentile (median), dotted red lines represent the 5th and 95th percentiles of the distributions surveyor sensitivity and specificity values were generated from. (TIF) [file pcbi.1012957.s031.tif]

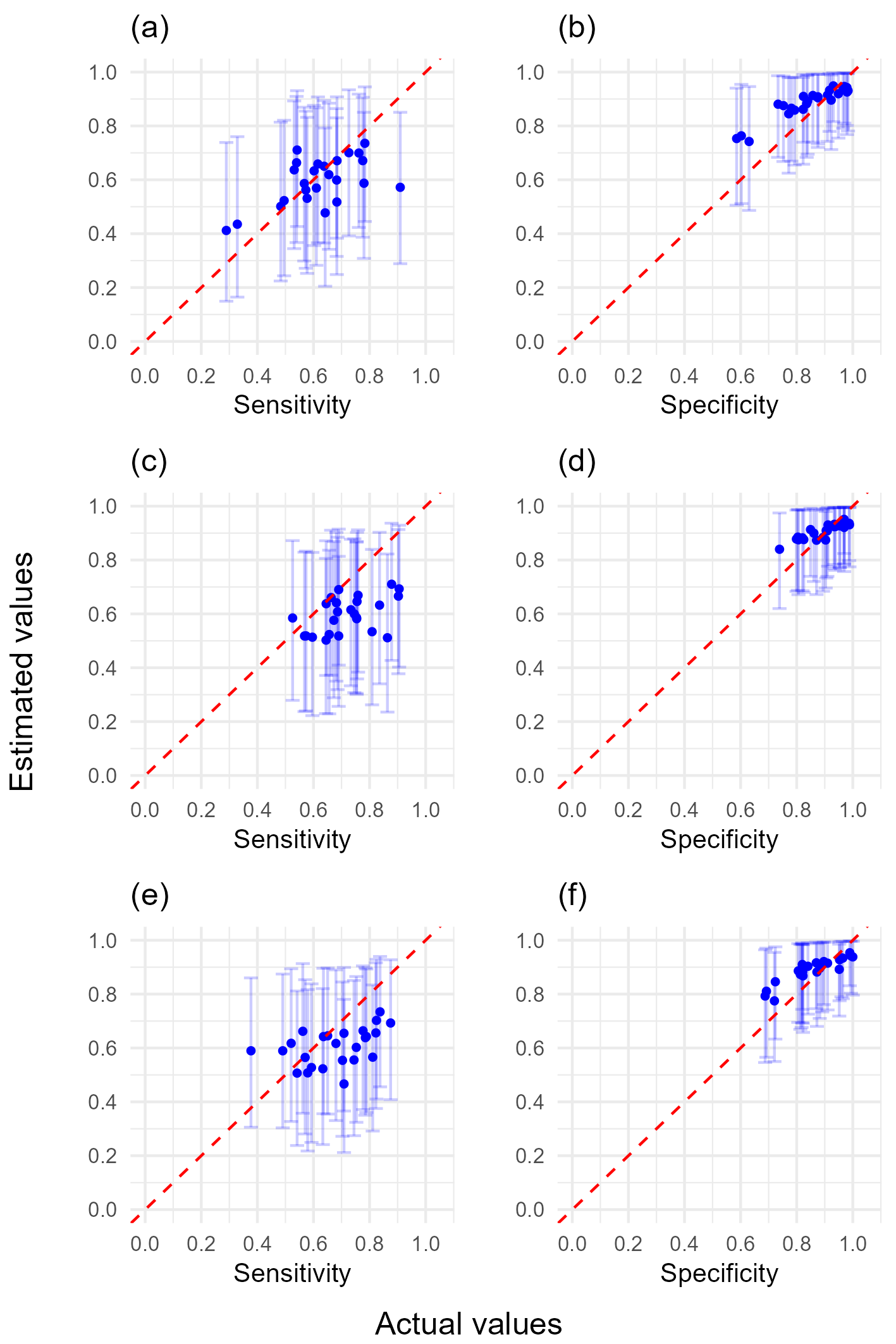

Supplement: S28 Fig — Good sensitivity and specificity prior distributions with misspecified prior knowledge which overestimated the true disease prevalence at survey locations, with surveyors assessing (a, b) 80, (c, d) 100, or (e, f) 120 trees in each of the higher and lower true disease prevalence locations. Error bars represent 95% confidence intervals, and dashed line represents perfect agreement between estimated values and actual values. (TIF) [file pcbi.1012957.s032.tif]
