# Supplementary material for: Unlocking plant health survey data: An approach to quantify the sensitivity and specificity of visual inspections
Source: PLoS Comput Biol. 2025 Nov 20;21(11):e1012957. doi: 10.1371/journal.pcbi.1012957 (PMC12671890; doi:10.1371/journal.pcbi.1012957)
Supplement: Attachment — Submitted filename: Response to Reviewers.pdf [file pcbi.1012957.s033.pdf]

Responses to reviewers and editors

Editors

We thank the editor for their positive assessment of the manuscript. We have not provided a user-friendly tool as requested by reviewer 4 because we believe it is not within the scope of this paper, with reasons detailed in response to comment 66. For this reason we have removed reference of a 'workflow' throughout the manuscript as suggested by the editor, instead describing the proposed methods as 'an approach', which is consistent with the title of the manuscript. We have also updated the shared code online and provided an updated DOI in the manuscript where the code is archived. This did not affect the running of the analyses to produce results for the manuscript (see Github and/ or Zenodo for details of changes).

2) Thank you for including an Ethics Statement for your study. Please include (i) The full name(s) of the Institutional Review Board(s) or Ethics Committee(s) (ii) The approval number(s), or a statement that approval was granted by the named board(s).

Response: Thank you for bringing this to our attention, we have now clarified this with the additional information that 'The data are part of a multi-annual project assessing accuracy of volunteer reporting of AOD at the study sites, approved by the Forest Research Ethics Board'. **See lines 166-168**.

Please confirm (a) that you are the photographer of 1, or (b) provide written permission from the photographer to publish the photo(s) under our CC BY 4.0 license.

Response: I confirm that I am the photographer of the images in Figure 1, and have now included this information in the figure caption. **See line 145**.

Figure 2. Please (a) provide a direct link to the base layer of the map (i.e., the country or region border shape) and ensure this is also included in the figure legend; and (b) provide a link to the terms of use / license information for the base layer image or shapefile. We cannot publish proprietary or copyrighted maps (e.g. Google Maps, Mapquest) and the terms of use for your map base layer must be compatible with our CC BY 4.0 license.

Response: We were unable to source available satellite imagery to mark the ~4 km survey routes on study sites. For this reason, Figure 2 has been removed. We did not feel that a map of the UK with study site locations indicated as points justifies a figure in the text, particularly given figures of additional model runs have been added to the main manuscript (Figures 8-9).

Please upload all main figures as separate Figure files in .tif or .eps format. For more information about how to convert and format your figure files please see our guidelines: https://journals.plos.org/ploscompbiol/s/figures. We notice that your supplementary Figures are included in the manuscript file. Please remove them and upload them with the file type 'Supporting Information'. Please ensure that each Supporting Information file has a legend listed in the manuscript after the references list.

Response: We have removed figures and supplementary figures from the manuscript file and uploaded these files separately as specified in the journal submission guidelines for revisions.

Reviewer 1

We thank the reviewer for their enthusiastic assessment of the manuscript, particularly their comments on the novelty and clarity of the work.

Reviewer 2:

We thank the reviewer for their positive assessment of the manuscript, and their comments on the clarity of the work and its importance. We are grateful for their comments and suggestions which have improved the manuscript.

Analyses of AOD survey data (only the section, not exact line numbers of reviewer comments on the original submitted manuscript were provided)

(1) Please, clarify how sensitivity and specificity were calculated using the expert assessor as a reference

Response: This is done. We have now added an additional equation to show how these are calculated as well as further text to clarify this in the methods. See **lines 170-180**.

(2) Although the analysis is described in general terms, a more detailed specification of the model would be helpful. In particular, the model equation should be provided, and the response variable explicitly stated. Based on the description, it is not entirely clear whether the response variable corresponds to a binary data or to a proportion given an aggregation. Clarifying this point would improve the reproducibility and interpretation of the results.

Response: We agree with the reviewer that this could be made clearer and thank them for highlighting this.

The model equations are now provided as equations 2 (**lines 189-191**) and 3 (**lines 193-194**). Binary data was used in statistical analyses with the response variable of models either the probability of a true positive for sensitivity models or the probability of a true negative for specificity model. This is now explicitly stated in the methods (**lines 185-188**). The code and data used for models is also provided with the manuscript.

(3) Since generalised linear models were used, it is assumed that the individual surveyor was included as a fixed effect. If the aim is to generalise beyond the specific set of surveyors included in the study, it might be more appropriate to model this factor as a random effect. Although a fixed effect specification may be reasonable, especially with a small number of surveyors, it would be desirable to briefly justify this choice.

Response: We thank the reviewer for highlighting this. We have now added text for further clarity in the methods **(lines 198-201)** to specifically state that the purpose of fitting an individual surveyor as a fixed effect rather than a random effect is to statistically analyse the impact of the individual surveyors in our study on sensitivity and specificity.

Methods to quantify sensitivity and specificity in the absence of a 'gold-standard' expert dataset (only the section, not exact line numbers of reviewer comments on the original submitted manuscript were provided)

(4) Could you please confirm whether "individual" in the context of the multinomial categories refers to each tree assessed by surveyors? This would help ensure we correctly interpret the structure of the model.

Response: We thank the reviewer for highlighting this. We have removed the term individual and added text to clarify that the multinomial categories refer to the number of observations in each of the four categories (**lines 220-224**).

(5) Please, clarify what is meant by 'test' in this context, as the term is not explicitly defined (e.g. "In this context, each test refers to the assessment of a specific symptom by a given surveyor")

Response: We agree with the reviewer and thank them for highlight this. We have now added text on **lines 217-218** to clarify this.

Conclusions (only the section, not exact line numbers of reviewer comments on the original submitted manuscript were provided)

(6) Although it is highlighted that the results can be applied to diseases with similar symptoms, it might be helpful to further discuss how the variability observed in sensitivity and specificity could influence the applicability to other diseases.

Response: We thank the reviewer for this suggestion and we have now added text in the conclusions (**lines 682-686**) to clarify that the results should be applied to surveyors with similar experience to those in our study, i.e. trained citizen scientists (given this likely influences sensitivity and specificity). We also mention that distributions of sensitivity and specificity should be used to incorporate uncertainty estimates of these parameters.

Reviewer 3:

(7) I did not fully understand it and I feel unconvinced that the basic tenet of the paper is fulfilled, i.e. that the sensitivity and specificity of visual inspections of disease symptoms by, e.g., citizens, can be quantified without a gold standard. I find this hard to believe. This lack of being convinced may be an error on my side, or perhaps a misinterpretation of the authors' intent, so in this review I will try and explain what I found not clear and where my skepticism comes from. Maybe I have simply misunderstood some of the language used by the authors and clarifications will address the issues. I hope the comments below will enable the authors to further elaborate and justify their conclusions in a revision.

Response: We thank the reviewer for their time and detailed consideration of the paper. Although these concerns were not raised with the other reviewers, we do believe the reviewer has correctly highlighted elements of the paper that were not fully clear. We have addressed these through the reviewer's specific comments below.

(8) May be shortened to: "Knowing sensitivity and specificity of visual inspection is critical ..." **Comment on line 25 in original submitted manuscript.**

Response: This is now amended in the manuscript (**lines 25-26**).

(9) "To address this" should refer to a problem, but the problem was not stated. **Comment on lines 26-27 in original submitted manuscript.**

Response: We agree with the reviewer. We have now amended the text to better emphasise the problem is that sensitivity and specificity of visual inspection are not routinely quantified, but this is critical for effective contingency planning and outbreak management (which we address in this study using empirical data and statistical analyses) (**lines 25-27**).

(10) "these trees" at this point the reader cannot understand whether these are the exact same trees or the same species of trees or trees in the same area. Please clarify. **Comment on line 29 in original submitted manuscript.**

Response: We agree with the reviewer. We have now clarified this in the text (**line 29**). We have also added that these trees were labelled (**line 28**) to provide further clarity for the reader that these were the exact same trees.

(11) Replace "these parameters" by "sensitivity and specificity" for clarity. **Comment on line 31 in original submitted manuscript.**

Response: We agree with the reviewer that this change improves the clarity of the manuscript. This is now amended in the manuscript (**line 33**).

(12) Suggest omitting "rarely available" or put it in comma's or brackets or use ", which is rarely available". **Comment on line 32 in original submitted manuscript.**

Response: We believe this change would take emphasis away from the rare availability of gold-standard datasets so we have left this in the current form.

(13) at this point it is unclear whether "number of symptoms" means the number of different symptoms that were assessed, or the number of leaves or branches with symptoms on a tree. **Comment on line 34 in original submitted manuscript.**

Response: We thank the reviewer for this suggestion and have now amended the text for clarity (**line 36**).

(14) Suggest writing “type of” symptoms rather than “symptoms”. **Comment on line 34 in original submitted manuscript.**

Response: We have now added text in the manuscript for clarity (**lines 34-35**).

(15) Here it is unclear whether one merely needs to know which site has relatively low prevalence and which has relatively high prevalence, or one needs to know the values (0.3 and 0.6) of prevalence. Importantly, readers need an intuition why, when using a Bayesian estimation framework, there would not be a need to know the actual prevalence of disease to estimate sensitivity and specificity of citizen observers as long as there are two sites differing in prevalence available with the only knowledge being available is that the sites differ (or perhaps one is higher the other is lower in prevalence). Giving the reader this intuition is critical (at least for me it is). I did not get this intuition from the present version. **Comment on lines 34-36 in original submitted manuscript.**

Response: We thank the reviewer for the comment which could cause confusion with some readers. We have now added text to consistently specify in the manuscript that we need reliable knowledge of the true disease prevalence on the relatively high or low true disease prevalence sites (i.e. not misspecified prior distributions). We do not provide exact values of the true diseases prevalences. As can be seen in Figure 3 we provide a distribution estimate that is not exactly 0.6 or 0.3 and could be described as ‘moderately narrow/ moderately broad’. The confidence of the prior knowledge is reflected by the shape of the prior distribution, and can be adjusted accordingly. Using the distributions we provide in the manuscript, we previously examined the impact of simulated true disease prevalences of ~0.3 and ~0.6 on the higher and lower true disease prevalence sites – but these varied by as much as ~0.1 depending on the simulation (we direct the reader to S3 File with the simulated prevalence values within the manuscript on **lines 336-337**).

In response to the reviewer query, we have now added additional runs to the manuscript with true disease prevalence estimates that fall at the tails of these prior true disease prevalence distributions (lower site ~0.4 (83.3 percentile of prior distribution), higher site ~0.8 (91.4 percentile of prior distribution); lower site ~0.1 (7.0 percentile of prior distribution), higher site ~0.4 (10.0 percentile of prior distribution)). As we specify in the manuscript we explore this despite our assumption of reliable prior knowledge of true disease prevalence on sites (**lines 315-337; lines 647-654)** (covered in our response to comment 46). Our results highlight that these misspecified true disease prevalence prior distributions (i.e. with true disease prevalence values at the tails of distributions) resulted in larger estimate errors of sensitivity and specificity. However, given the size of the misspecification of the prior distribution, models were still reasonably robust (with the exception of extreme low disease prevalence which may be due to insufficient data for reliable Bayesian inference).

Overall, our results show that a reliable (but not precise) estimate of the prior distributions of true disease prevalence on sites is sufficient for Bayesian inference to estimate sensitivity and specificity of tests by providing direction for the model. By using two diagnostic tests in the presented equations, this provides narrower solutions within this estimated range of a high and true disease prevalence site for what the potential sensitivity and specificity of tests could be (covered in response to comment 54).

We have added text to the methods (**lines 315-337**), results (with figures) (**lines 460-500**), and discussion (**lines 612-620; 647-654**) that address this. We have also amended the abstract (**line 38**) for clarity. We have added data from these additional runs to S3 File, and plotted results in **Figures 8-9**, and **S19-28 Figures**. We have adjusted the wording of text accordingly throughout (including in captions for **Figures 6-7; S2-S18 Fig**) for clarity given we now run different scenarios of true disease prevalence prior knowledge. Given that the output Figures of new model runs label either 'sensitivity' and 'specificity' on all panels in the plots, we have also added these labels to for the previously presented outputs (**Fig 6-7; S2-17 Fig**).

(16) "these trees" It is not clear whether this means the exact same individual trees or the same species of tree. **Comment on line 51 in original submitted manuscript.**

Response: We have now added text to clarify this in the manuscript (**line 54**).

(17) "these parameters" -> sensitivity and specificity. **Comment on line 54 in original submitted manuscript.**

Response: We agree with the reviewer that this change improves the clarity of the manuscript, and have adjusted the text (**line 58**).

(18) omit "rarely available" and add it at the end ", which is rarely available". **Comment on line 55 in original submitted manuscript.**

Response: We believe this change would take emphasis away from the rare availability of gold-standard datasets.

(19) is the impact of 5 billion euro per year or is it accumulated (and potentially discounted) over a certain period? **Comment on line 70 in original submitted manuscript.**

Response: We thank the reviewer for highlighting this. The stated 5 billion impact is the upper estimate of an accumulated impact over a 50 year period. This is now clarified in the text on **line 75**.

(20) Suggest to add common names for the disease and the tree species. **Comment on lines 71-76 in original submitted manuscript.**

Response: We agree with the reviewer that adding common names for the disease and tree species improves the clarity of the text. We have added this on **lines 79-81** with an additional required reference for this:

[9] Kowalski T. Chalara fraxinea sp. nov. associated with dieback of ash (Fraxinus excelsior) in Poland. Forest Pathology. 2006;36(4):264-70. doi: https://doi.org/10.1111/j.1439-0329.2006.00453.x.

(21) such -> introduced (or alien or invasive) (for clarity). **Comment on line 77 in original submitted manuscript.**

Response: We agree with the reviewer that adding this improves the clarity of the text and have now done this on **line 83**.

(22) suggest to omit "strategies". **Comment on line 80 in original submitted manuscript.**

Response: We agree with the reviewer that the word "strategies" is not necessary and have now removed this from the manuscript (**line 86**).

(23) suggest to start new paragraph here to improve readability. **Comment on lines 85-86 in original submitted manuscript.**

Response: We thank the reviewer for this suggestion, and agree that it improves the readability of the manuscript. This is now amended (**lines 91-92**).

(24) This text is clearer than previous text stating an expert had monitored these trees. **Comment on lines 101-102 in original submitted manuscript.**

We thank the reviewer for drawing our attention to this.

(25) Suggest to start a new paragraph at "Next". **Comment on line 102 in original submitted manuscript.**

Response: We thank the reviewer for this suggestion, and agree that introducing new paragraphs improves the readability of the text. We have now broken up the text at the end of the introduction to improve the readability of the manuscript as well as incorporating the literature highlighted by reviewer 4 (**lines 100-125**).

(26) see previous comment on "these parameters". **Comment on line 103 in original submitted manuscript.**

Response: We agree with the reviewer that specifically stating sensitivity and specificity rather than "these parameters" improves the clarity of the text. We have now amended this (**line 118-119**).

(27) Use full name Acute Oak Decline (Abbreviations not suitable in title). Line 103 is perhaps a proper place to introduce the abbreviation AOD after the full term. **Comment on lines 112-113 in original submitted manuscript.**

Response: We thank the reviewer for highlighting the use of an abbreviation in a subheading. We have now replaced 'AOD' in all subheadings with 'acute oak decline'. We have not defined the abbreviation AOD at the suggested line number (assuming the reviewer means the first

sentence of the methods section) because we have already defined AOD at first mention in the main text on **line 112**.

(28) Add common names perhaps? **Comment on line 114 in original submitted manuscript.**

Response: We thank the reviewer for this suggestion, and agree that adding the common names of the oak species increases the clarity of the text. We have amended this on **lines 131**.

(29) Not sure what OS grid reference is. Can you add information or a reference how readers can use this to locate the sites? **Comment on line 146 in original submitted manuscript.**

Response: We thank the reviewer for highlighting this and have amended the text to now provide the grid reference of sites in latitude, longitude format (**lines 148-149**).

(30) Since 2019 and 2010. **Comment on line 149 in original submitted manuscript.**

Response: We agree with the reviewer that adding the word "since" improves the clarity of the text. We have now amended the text (**line 151-152**).

(31) I suppose you mean that 20 of the 23 volunteers had experience with tree work as well as knowledge of AOD, but I am not entirely sure, given the way it is formulated. **Comment on lines 168-170 in original submitted manuscript.**

Response: We are grateful to the reviewer for highlighting this. The original text was a typographical mistake that is now corrected (**lines 157-159**). Nine individuals had worked as professional tree surveyors, and only three declared no knowledge of AOD. The purpose of the text is to emphasise the prior knowledge base of the citizen scientists in relation to trees. The full questionnaire and results are provided in S1 File.

(32) I suppose you mean that sensitivity and specificity of the detection of EACH separate symptom was assessed. Please clarify as such so readers are not in doubt. **Comment on line 181 in original submitted manuscript.**

Response: We thank the reviewer for highlighting this, and agree that this is not clear in the original text. We have now amended the text to clarify that the sensitivity and specificity of the detection of each separate symptom was assessed (**lines 171-172**).

(33) Did you make one overarching model in which the type of symptoms was a covariable or did you analyse data separately for each symptom? **Comment on lines 183-185 in original submitted manuscript.**

Response: We agree with the reviewer that the model described in the original text needs extra clarity. The type of symptom was a covariable. As requested by reviewer 2, we provide the models described in equations 2 and 3 for clarity (**lines 189-191; lines 193-194**).

(34) logit link function. **Comment on line 185 in original submitted manuscript.**

Response: We thank the reviewer for highlighting this, and have now amended the text (**line 183**).

(35) I cannot get my head around the statement that using Bayesian estimation, the sensitivity and specificity of diagnostic tests can be estimated when two or more tests are applied to the same individuals across two or more populations with different true disease prevalences without knowledge on the actual prevalence in these areas. This is a premise that is fundamental to the paper and I feel it warrants more text to help the reader get an intuition. I am aware that references are provided, but this principle is so important that I think it needs elaboration in the paper itself. And I am doing this review on an intercontinental flight without the benefit of the internet. **Comment on lines 194-196 in original submitted manuscript.**

Response: We agree with the reviewer that this is a central tenet of the paper and is worth emphasising and discussing further. We have now directed the reader to the equations which provide the logic to this statement (**lines 209-211**) and have added further text to the discussion to explain this (**lines 612-620**). Further detail to this query is also given in response to comments 15 and 54.

(36) The way this is written you seem to say that these methods assume independence of sensitivity and specificity, but is that possible at all? Or perhaps you mean independence of the test result for different tests on the same individual, which should be unlikely if the tests are proper? Please clarify. **Comment on line 197 in original submitted manuscript.**

Response: We thank the reviewer for highlighting this. The wording in the manuscript has now been amended to clarify that the methods assume independence between both the sensitivity of different tests and between the specificity of different tests, rather than between sensitivity and specificity (**lines 208-209**). We have amended wording throughout the manuscript when referring to covariance or independence/ non-independence to ensure that this is clear.

We also refer the reader to an additional supplementary file that we have added which provides further explanation of covariance parameters used in models (**S4 File**) and refer the reader to this when detailing methods (**lines 212-213; line 232**). We also added text to provide further clarity (**lines 211-212**) to explain that '*such non-independence arises when tests measure comparable biological processes of a disease that are therefore likely to have associated test errors*', and provide a citation for this (Branscum et al., 2005) whilst directing the reader to further detail on independence/ non-independence between tests in the discussion and S4 File.

(37) The way this is written it seems you do not use this non-independence, but I think you do, don't you? **Comment on lines 197-198 in original submitted manuscript.**

Response: We use both non-independence and independence models and believe this is clear in the manuscript. We have added text to **lines 213-214** for added clarity that we are referring to both independence and non-independence models.

(38) "We apply these models" Which ones? With or without assumed independence? **Comment on line 200 in original submitted manuscript.**

Response: We thank the reviewer for highlighting that this is unclear and now state that we apply "both" models (**line 215**).

(39) Here a method is elaborated for two tests (i.e. two symptoms). But in the case study you had three symptoms. It would be good to mention in the very beginning that the theoretical case (simulations) is elaborated for two symptoms, not three as in the case study. I only realized this later. **Comment on lines 202-208 in original submitted manuscript.**

Response: We thank the reviewer for highlighting this, and have now added text at the end of the opening paragraph of the method section 'Methods to quantify sensitivity and specificity in the absence of a gold-standard' expert dataset' that highlights this (**lines 218-219**).

(40) I am puzzled by reading about separate covariances for disease positives and disease negatives. Usually, we have a covariance between values of two variables, irrespective of the values. Here there are two covariances, one for negative outcomes of two tests and another for positive outcomes. Is this still the common definition of covariance or is it a different thing altogether? Maybe this is better explained in 245-247, but then I am already confused. **Comment on lines 217-222 in original submitted manuscript.**

Response: The two variables here are sensitivity between the two tests (covariance between tests for a diseased host) and specificity between the two tests (covariance between tests for a non-diseased host), and have added text to clarify this (**lines 231-232**). The equations for calculating this are provided in the manuscript. We now provide further information in an additional supplementary material (**S4 File**) and direct the reader to this (**lines 212-213 and 232**). The covariances values represent the quantity and direction of related detection probability between tests for disease positives and tests for disease negatives.

(41) Do you report the minimum and maximum values? **Comment on line 238 in original submitted manuscript.**

Response: This is provided the supplementary materials (S3 File), and we have now amended the text later in the methods to make this clearer (**line 280**).

(42) This sentence is hard to understand. You are calculating something (covariances) that was assumed in the first place, but you don't say why. Is this to find out how well you could estimate covariances from the generated data? What were the minimum and maximum values? That is still based on the case study? So, to what extent is the simulation exercise based on the case study? **Comment on lines 254-257 in original submitted manuscript.**

Response: We thank the reviewer for highlighting this and agree that further text is required for clarity. We calculated the covariance values for simulated datasets because we didn't simulate

a specific covariance value, but rather a probability of 0.85 that symptom two would be scored positive if symptom one was scored as positive and the host was disease positive and that symptom two would be scored as negative if symptom one was scored as negative and the host was disease negative.

The covariance and potential range of covariance value in each run will vary depending on surveyor sensitivity and specificity as can be seen from equations 8 and 9, therefore we believed it important to calculate these values for simulated datasets and provide them in the stated supplementary material (S3 File) to give better understanding of the size of this value in each model run. We have added further text in **lines 280-283** for clarity.

(43) This is hard to follow. It might be helpful to provide a mathematical derivation of these metrics, starting at theory that most readers are probably familiar with. It could be in SI if it is unwieldy. **Comment on lines 258-268 in original submitted manuscript.**

Response: We thank the reviewer for highlighting that different presentation of equations and additional clarity are required. We have now amended the equations for the covariance parameters (**lines 286-289**), and explained their derivation in the additional supplementary file (**S4 File**) which we direct the reader to in the text (**lines 282-283**).

(44) Fig 2. Caption should state what is given by the curve with the purple area under the curve (priors?). Then the dashed distributions are the assumed parameter distributions for generating data for analysis?

Response: We thank the reviewer for highlighting this and have now amended the caption to provide this information (**lines 299-303**).

(45) How close to the case study are we? Are you using actual data on bleeds, cracks and exit holes? **Comment on lines 290-291 in original submitted manuscript.**

Response: We agree with the reviewer that this required extra clarity and have added additional text (**lines 306-310**). We simulated data from the distribution of data on detecting stem bleeds. We only used the distributions for stem bleeds rather than the other symptoms because our results show that this was a reasonably reliable symptom to inspect for when accounting for both false positives and false negatives – so we deemed this realistic for organised pest/ disease surveys.

(46) Please provide context for your anticipation. This is your expectation about what data might be available in practice? Do you expect that data will be available to estimate priors for prevalence in a high and low prevalence area? **Comment on line 292 in original submitted manuscript.**

Response: We thank the reviewer for raising this uncertainty and have now amended the text to provide further clarity. In practice, it is a reasonable assumption to estimate the true disease prevalence within the accuracy that we have examined within the manuscript using a combination of local expert knowledge of sites and using statistical methods that utilise

epidemiological knowledge of the pest. In our paper we specifically highlight the simple 'rule of thumb' approach to estimate true disease prevalence following detection of a pest with knowledge of the epidemic growth rate when a fixed number of samples (or observations) are taken at known regular intervals. We provide the reference for this in the text (Parnell et al., 2015. Early detection surveillance for an emerging plant pathogen: a rule of thumb to predict prevalence at first discovery. *Proc. R. Soc. B* 282: 20151478. http://dx.doi.org/10.1098/rspb.2015.1478). We clarify this on **lines 316-322; 612-620; 647-654**.

We have also added additional model runs and text to the manuscript to examine the sensitivity of outputs to misspecified true disease prevalence prior distributions (addressed in comment 15) which can be avoided through combining local expert site knowledge with statistical approaches that account for pest epidemiology, and using appropriate methods to specify priors (**lines 647-654**).

(47) Prior knowledge: add 'on prevalence'. **Comment on line 293 in original submitted manuscript.**

Response: We thank the reviewer for highlighting that the text needed additional clarity here, and have added text (**line 317**).

(48) How do you know the minimum and maximum possible values? **Comment on lines 298-299 in original submitted manuscript.**

Response: We thank the reviewer for their query. We have provided this information in equation 9, earlier in the manuscript (**lines 291-294**) and we direct the readers to this in the text. We have amended the text to also direct the reader to an additional supplementary file (**S4 File**) that provides further detail on the covariance parameters (**line 282-283**).

(49) "as described previously" might be replaced by an actual list of parameters estimated. **Comment on line 321 in original submitted manuscript.**

Response: We thank the reviewer for highlighting that this is not clear in the manuscript. We have now amended the text to remove 'as described previously', and state that we 'iterate across a range of values for each model parameters' which we believe provides clarity for the reader whilst remaining concise (**lines 354-355**).

(50) Better use full name for title. **Comment on line 341 in original submitted manuscript.**

Response: We have adjusted the text to use the full term 'acute oak decline' in section headings rather than 'AOD' throughout the manuscript and thank the reviewer for bringing this to our attention.

(51) How is the frequency of symptoms defined? Is frequency the same as prevalence? If so, use one term. **Comment on line 346 in original submitted manuscript.**

Response: We thank the reviewer for highlighting that this requires extra clarity and have now amended the text (**line 380**). Frequency is the number of times the symptom appears on an individual tree.

(52) These are interactions in the glm? Maybe use the term "dependency"? **Comment on line 346 in original submitted manuscript.**

Response: We thank the reviewer for highlighting that the model structure is not clear from the results. We now provide these in **equations 2 and 3** in the manuscript (**lines 189-191; lines 193-194**)

(53) Fig. 4: what is the reason that the CI is different for surveyors having similar sensitivity? Why is there less certainty on the performance of some surveyors than others? Did they sample fewer trees?

Response: Some surveyors sampled fewer trees and this information is available in S2 File. No changes have been made.

(54) But does having such information not qualify as having a (gold) standard? Or is that just a silver or bronze standard? Maybe I misread the article because of misinterpretation of the term "gold standard". Even if you don't have exact information on the disease status of each and every tree, as long as you have a distribution (and an average), you can estimate how good a test is (though there is a chance of false positives making up for missed true positives). There is perhaps also a more fundamental issue here. In normal Bayesian inference, we estimate a parameter for which we have a prior. So we estimate a posterior, given the data, to update the prior. But it is still the same variable. Here, the prevalence is uncertain (but a prior is available), and you estimate test results, where some positives may be missed and some negatives may be mislabeled. Then, the result of analysis is another estimate of prevalence, and sensitivity and specificity are then probably (is my understanding) still calculated as if the priors can be used as a "standard". Is my assumption right or am I wrong? How not-gold is such a standard? **Comment on line 402 in original submitted manuscript.**

Response: We have now defined gold-standard at first mention in the main manuscript as a diagnostic test with perfect detection sensitivity and specificity (**lines 101-102**), as well as in the abstract (**lines 31-32**) and author summary (**line 59**). This is consistent with previous literature.

The equations that we provide for Bayesian modelling (Equations 4, 5, 6) do not assume perfect knowledge of any parameter so would not qualify as a gold-standard. We provide probability estimates of the distributions of the different parameters in the model (prior distributions) and then given the observed data (and our prior distributions), we use Bayesian inference to estimate the probability densities of parameters given the observed dataset (the posterior distributions of parameters), i.e. at what probability would particular parameter combinations occur with the survey observed dataset given our prior distributions (equation 7). At each iteration of the MCMC chain, different parameter values are sampled, and we use these chains for the posterior probability estimates of model parameters – when chains did not adequately

converge to a value results were discarded because this suggests that the model was not identifiable given the data and prior information. This is detailed in the methods section.

We express reliable prior knowledge of the true disease prevalence on sites that have a relatively higher or lower true disease prevalence (with logic for this now clarified in the manuscript and provided in response to comment 46). As highlighted in response to comments 15 and 46, we also provide additional model runs with misspecified prior distributions of true disease prevalence on sites to examine the impact on output estimates.

Our results support that reliable prior distributions of true disease prevalence (i.e. not misspecified prior distributions with values at the tails of prior distributions) provides sufficient information for Bayesian inference of parameters by using data for two diagnostic tests on the same hosts on each of the different prevalence locations with informative prior knowledge of two model parameters (true disease prevalences in our instance) (in addition to specifying minimum and maximum possible covariance parameter values). Models that use only one diagnostic test, or are conducted on only one site are not identifiable because they provide much less information for inference. This means that they are dependent on the prior probability estimates and not Bayesian inference. For example, using one site and one diagnostic test, this would be written as:

$$Probability\ test\ positive = \pi Se + (1-\pi)(1-Sp)$$

There are a number of combinations of π, Se, and Sp which could be used to solve this for any given probability. It is evident that the equations we provide in the manuscript provide much more data for inference than the above.

We have added text in the discussion to further emphasise this (**lines 612-620; lines 647-654**). We have also added text in the abstract when outlining results (**line 40**) to emphasise that Bayesian modelling was used. We believe that the methods in the manuscript sufficiently outline this process, with reviewers 1,2 and 4 commenting on the clarity of the work.

(55) hinders. **Comment on line 474 in original submitted manuscript.**

Response: We thank the reviewer for highlighting this typographical error. We have now adjusted ‘hinder’ to ‘hinders’ in the manuscript (**line 506**)

(56) Rephrase. Now it says” Ability between [...] different symptoms. I get your meaning, but reads awkward. **Comment on line 476-477 in original submitted manuscript.**

Response: We agree with the reviewer that extra clarity is required and we have amended the text in the manuscript (**line 508-509**).

(57) Omit “rarely available” Insert “lack of” before “informative prior information”. **Comment on line 480 in original submitted manuscript.**

Response: We thank the review for this suggestion and have amended the text accordingly (**line 512-513)**.

(58) Add "required" after "assessments". **Comment on line 483 in original submitted manuscript.**

Response: We agree this change adds clarity to the text and have amended this in the manuscript (**line 515**).

(59) It is not obvious how you have used your data to conclude that "particular symptoms will influence optimal survey locations". Please explain. **Comment on line 490 in original submitted manuscript.**

Response: We thank the reviewer for their comment and have added additional text for clarity (**lines 523-527**). Earlier in the paragraph we mention that the sensitivity of a detection method affects the optimal deployment of surveillance strategies for early detection of a pest or disease in the landscape, with lower sensitivity leading to surveillance focussed in higher-risk locations (**lines 517-520**). The added text specifically outlines how this would apply to our data (**lines 523-527**) e.g. exit holes are associated with a lower sensitivity of detection than stem bleeds, which means that early detection surveillance when assessing exit hole symptoms are more likely to be focussed around higher-risk locations than when assessing stem bleed symptoms, which are more likely to be further spread in the landscape.

(60) I'd say "an Agrilus species" not another Agrillus species as EAB is the only Agrilus species that has killed millions of ash trees in the USA. **Comment on line 495 in original submitted manuscript.**

Response: We thank the reviewer for this suggestion and have now amended this in the text (**line 538**).

(61) are directly comparable to -> "are similar to" or "resemble" (also in 498). Insert "the" before A. biguttatus. **Comment on line 496 in original submitted manuscript.**

Response: We thank the reviewer for their suggestion, but we have not amended the text. Directly comparable here infers that the exit holes are more than similar and more than resemble each other. Both exit holes are D-shaped and approximately 3-5 mm in diameter.

(62) abundance is the same as frequency, a word used earlier in the text? **Comment on line 503 in original submitted manuscript.**

Response: We thank the reviewer for highlighting this, and we have now amended the text to 'frequency of a symptom on a tree' to provided consistency through the manuscript (**line 546**).

(63) Suggest stating: "All citizen observers involved in the study …" **Comment on line 508 in original submitted manuscript.**

Response: We agree that this improves the clarity of the text and have now adjusted for this in the manuscript (**line 552**).

(64) Species –> parasitoid (damage scores are not a species). **Comment on line 518 in original submitted manuscript.**

Response: We have not amended the text because we were not referring to damage scores, but counts of the species (**lines 564-567**).

(65) The word "envisage" is not so clear. You mean "recommend"? To whom? To achieve what? **Comment on line 550 in original submitted manuscript.**

Response: We thank the reviewer for their suggestion. We have now amended text to explicitly state that the approach described can be applied by plant health authorities (**line 624**).

Reviewer 4:

We thank the reviewer for their comments on the clarity and novelty of the work. We are grateful to the reviewer for drawing our attention to the literature from Australasia, which we have now to the manuscript to further improve the strength of the manuscript.

(66) My only real reservation is that after looking at the supplied code, the touted "workflow" for estimating sensitivity and specificity is not really approachable except by experts. It would be great if they could supply something more user-friendly like a tool, maybe with a structured Excel file for practitioners to enter how many plants were sampled, by who, and what was observed. Then a script would suck that in and spit out the results. This may be asking too much, but it is what I thought of when I saw "workflow".

Response: We agree with the reviewer that a user-friendly tool would aid the use of the work. Such a tool was provided to the funders of the work but in a private capacity and tailored to their needs. Although this is not available to the reader, we have made the code available and have added text to the discussion to describe how this could developed and tailored for the specific needs of a reader. We believe that many modelling studies would benefit from provision of a generalised user friendly tool, but this can be technically demanding and is not within scope of the paper.

To avoid confusion that this is being delivered by the manuscript, we have now avoided the word workflow, and changed it to the term 'approach' throughout which is consistent with the title. In this instance, we believe that a user should have good knowledge of the 'ins and outs' and potential pitfalls (such as prior probability misspecifications) of the methods. Such application is therefore probably best done in conjunction with parties with statistical knowledge to tailor the approach to their specific problem. The code we provide and the methods detailed will facilitate this.

(67) I have few other suggestions, except for a criticism that, based on some written statements and the rather Euro-centric citations, the authors seem unaware of a lot of relevant work from Australasia and North America. I have some sympathy with the authors as I am acutely aware that the sheer volume of research is now such that it is impossible to be familiar with it all. But in this case, it renders some of their statements inaccurate, including that they are the first to estimate sensitivity and specificity of visual inspections for plant disease in the absence of a gold standard. More details below...

Response: We thank the reviewer for bring this to our attention and we have conducted an additional literature search and addressed this throughout the manuscript.

We have amended the introduction and discussed the below manuscripts in the introduction (**lines 102-108**), and the discussion (**lines 527-534; lines 556-563; line 576-577; line 582**) using the below references which we believe add strength to the manuscript:

[19] Mangano P, Hardie D, Speijers J, Johnston R, Sousa-Majer M, Maynard G. The Capacity of Groups within the Community to Carry out Plant Pest Surveillance Detection. The Open Entomology Journal. 2011;5:15-23. doi: 10.2174/1874407901105010015.

[20] Esfandi K, Afsar S, Richards K, Hedderley D, Brown SDJ, Najar-Rodriguez A, Ormsby M. Determining the efficacy of visual inspections at detecting non-biosecurity–compliant goods. Journal of Vision. 2024;24(8):8-. doi: 10.1167/jov.24.8.8.

[21] Bulman L, Kimberley M, Gadgil P. Estimation of the efficiency of pest detection surveys. New Zealand Journal of Forestry Science. 1999;29:102-15.

[22] Pirtle EI, Umina PA, Hauser CE, Maino JL. Using mock surveillance to quantify pest detectability prior to establishment of exotic leafminers. Crop Protection. 2021;148:105713. doi: https://doi.org/10.1016/j.cropro.2021.105713.

[23] Hammond NEB, Hardie D, Hauser CE, Reid SA. Can general surveillance detect high priority pests in the Western Australian Grains Industry? Crop Protection. 2016;79:8-14. doi: https://doi.org/10.1016/j.cropro.2015.10.004.

[45] Caley P, Welvaert M, Barry SC. Crowd surveillance: estimating citizen science reporting probabilities for insects of biosecurity concern. Journal of Pest Science. 2020;93(1):543-50. doi: 10.1007/s10340-019-01115-7.

[48] Johnson KA, Brannen PM, Chen C, Bock CH. Visual Assessment of Phony Peach Disease: Evaluating Rater Accuracy and Reliability. Plant Disease. 2024;108(4):930-40. doi: 10.1094/pdis-11-22-2669-re.

[52] Arndt E, Rumpff L, Lane S, Bau S, Mebalds M, Kompas T. Estimating probability of visual detection of exotic pests and diseases in the grains industry—An expert elicitation approach. Frontiers in Ecology and Evolution. 2022;Volume 10 - 2022. doi: 10.3389/fevo.2022.968436.

We also add reference to a manuscript in the conclusions which outlines how expert data (which may be observation data) is required for ground truthing remote sensing, but is not currently known how accurate this is (**lines 702-704**):

[54] Fassnacht FE, White JC, Wulder MA, Næsset E. Remote sensing in forestry: current challenges, considerations and directions. Forestry: An International Journal of Forest Research. 2023;97(1):11-37. doi: 10.1093/forestry/cpad024.

(68) I suggest replacing "usually" with "often" since non-visual sampling methods are the norm for non-pathogen pests. **Comment on line 82 in original submitted manuscript.**

Response: We thank the reviewer for this suggestion and have amended the text in the manuscript accordingly (**line 88**).

(69) Contrary to this statement, Cardwell et al. 2018 (https://apsjournals.apsnet.org/doi/10.1094/PHP-06-18-0036-RV) write "There are hundreds of diagnostic plant disease assays used every day in the United States that either are not validated or are validated in an ad hoc way." Does this indicate a difference between Europe and America? **Comment on line 86-88 in original submitted manuscript.**

Response: We thank the reviewer for their comment. We have now amended the wording of the text for clarity and added the suggested reference to strengthen our statement. The paper highlighted by the reviewer highlights the requirement to validate regulatory diagnostics in the United States, whilst the reference we originally provide from Europe outlines the requirements for laboratories preparing accreditation for a plant pest diagnostic activity https://onlinelibrary.wiley.com/doi/full/10.1111/epp.12780. These manuscripts support the amended text that 'although the importance of quantifying the reliability of laboratory diagnostic tests is recognised (e.g. through accreditation schemes or requirements to validate regulatory diagnostics), the probability of detecting a pest via initial inspection is rarely quantified.' (**lines 92-95**).

(70) This is not true in Australasia, where the probability of detecting a pest via initial visual inspection has been studied a lot, admittedly with relatively few of those studies being published. Some citeable examples include: Bulman et al. 1999 (http://www.scionresearch.com/__data/assets/pdf_file/0004/17248/NZJFS291_1999_102_115BULMAN.pdf), Froud et al 2008 (https://nzpps.org/_oldsite/books/2008_Surveillance/Surveillance.pdf), Hauser & McCarthy 2009 (http://onlinelibrary.wiley.com/doi/10.1111/j.1461-0248.2009.01323.x/abstract), Mangano et al. 2011 (https://benthamopenarchives.com/abstract.php?ArticleCode=TOENTOJ-5-15), Hester & Cacho 2012 (http://www.tandfonline.com/doi/abs/10.1080/10807039.2012.632307), Hammond et al. 2016 (https://doi.org/10.1016/j.cropro.2015.10.004), Caley et al. 2020 (http://link.springer.com/10.1007/s10340-019-01115-7). **Comment on line 88-89 in original submitted manuscript.**

Response: We have now amended the text throughout and added relevant references to acknowledge relevant previous work (see response to comment 67). We maintain however that aside from these studies, this is not standard practice in plant health.

(71) It is not clear to me what is meant by "test one" and "test two". It feels like a little more explanation is needed to set up here. **Comment on line 203 in original submitted manuscript.**

Response: We thank the reviewer for highlighting this. We have now added text to clarify this (**lines 217-218**).

(72) I suggest pointing out that the priors are shown in purple. **Comment on line 278 in original submitted manuscript.**

Response: We thank the reviewer for highlighting this. We have now amended the caption (**lines 299-303**).

(73) The authors will be disappointed to learn that their study is not the first to derive quantitative estimates of the sensitivity and specificity of visual inspection for symptoms in plant health in the absence of a gold-standard reference dataset: see Chapter 4 in Froud et al 2022 (https://www.knowledgeauckland.org.nz/media/2398/tr2022-08-2021waitakere-ranges-kauri-population-health-survey06chapter-4.pdf). However, the simulation exploration, workflow and R code of the current study are very welcome enhancements. **Comment on lines 471-473 in original submitted manuscript.**

Response: We thank the reviewer for bringing this to our attention. We have now added reference to this work within the manuscript in the introduction (**lines 104-108**) and the discussion (**lines 600-609**), whilst also detailing how our study builds on this work.

(74) See Mangano et al. referenced above. **Comment on lines 510-511 in original submitted manuscript.**

Response: We thank the reviewer for the suggested reference and have added this into the manuscript as mentioned above in response to comment 67 (**lines 103, 529, 533, 559, 582**).
