# Supplementary material for: Unlocking plant health survey data: An approach to quantify the sensitivity and specificity of visual inspections
Source: PLoS Comput Biol. 2025 Nov 20;21(11):e1012957. doi: 10.1371/journal.pcbi.1012957 (PMC12671890; doi:10.1371/journal.pcbi.1012957)
Supplement: Attachment — Submitted filename: R2_Response to reviewer.pdf [file pcbi.1012957.s034.pdf]

Reviewer comments

(General comment) Authors have made an effective revision that results in a much clearer manuscript (at least for this reader). Upon reading the revision, I still found a few things less clear, and these are pointed out below. I think it is easy to fix and it is at the discretion of the authors as far as I am concerned. It is very interesting work, and I feel it will be welcomed in the community, though I suspect many people will find it difficult to understand. Hence, there remains a need to try and be as clear and explicit as possible to avoid readers getting lost.
Response: We thank the reviewer for their positive and helpful comments on the manuscript and highlighting areas where the text is unclear. We have amended these in the manuscript where appropriate (see below) to improve the clarity of the text and provided the line numbers for these changes in the revised manuscript where they are also marked-up with red text. We also provide the line numbers of the reviewer comments on the previously submitted manuscript.

We have also corrected typographical errors on **line 155** (capitalising word); **line 351** (changed word to singular); **lines 503, 1111, 1129, 1147, 1168, 1186** (removed repeated word); **line 637** (changed verb conjugation).

(1) "using simulated data" Readers may wonder "simulated data for WHAT"? It might help if this could be stated explicitly. **Comment on line 33 in the previously submitted manuscript.**
Response: We thank the reviewer for their helpful comment, and we have now provided clarity on what the data are and what they are used for by rephrasing this sentence and adding additional detail. We have now amended the sentence to read '*The utility of an approach using Bayesian modelling to estimate the sensitivity and specificity of visual inspection in the absence of a rarely available 'gold-standard' dataset was then examined with simulated plant health survey datasets*'. This now provides the reader with information that we used simulated plant health survey datasets to test the approach using Bayesian modelling to estimate the sensitivity and specificity of visual inspection in the absence of a rarely available 'gold-standard' dataset. We are unable to add further detail here in the abstract due to word limitations (currently on the maximum 300 words). **Changes made lines 32-34.**

With this in mind, we have also amended figure captions for additional clarity on what the simulated data were. These captions previously referred to '*Sensitivity and specificity distribution output using simulated data …*', but have now been amended to read '*Sensitivity and specificity distribution output using simulated survey datasets …*' **Changes made lines 466; 499; 954; 973; 994; 1016-1017; 1036-1037; 1057-1058; 1079-1080; 1105-1106; 1125-1126; 1143-1144; 1162-1163; 1182-1183.**

In the abstract, to avoid confusion we have also replaced the word '*significant*' with '*substantial*' in the sentence '*Invasive plant pests and pathogens cause significant environmental and economic damage*' given we are not referring to the outcome of a statistical test here. **Change made line 22.**

(2) "this makes it" -> "it is". **Comment on line 97 in the previously submitted manuscript.**
Response: We thank the reviewer for this suggestion and have amended the text accordingly. **Change made line 98.**

(3) These sentences are very vague. Readers may really be helped if you state in words what you do (conceptually) before getting into the details (as you do in the methods). This would help them getting their bearings before it gets more mathematical (and many readers will skip). You want that those readers can still correctly interpret the figures. This would be helped by stating clearly that you assess sensitivity and specificity of individual assessors and give readers an intuition how that is achieved. **Comment on lines 118-120 in the previously submitted manuscript.**
Response: We thank the reviewer for highlighting that an overview of the methods to estimate the sensitivity and specificity in the absence of gold-standard data at the end of the introduction section would be beneficial for understanding the methods section. We have now added a brief overview of methods for the reader prior to the detailed methods section. This new text is now in a new paragraph to break up the text. **Changes made lines 120-133.**

(4) Spelling: buprestid. **Comment on line 130 in the previously submitted manuscript.**
Response: We thank the reviewer for noticing this and have now amended this in the text. **Changes made line 143.**

(5) There is only a single bleed on the photo. Revise text accordingly. **Comment on Fig 1a caption (Line 143) in the previously submitted manuscript.**
Response: We thank the reviewer for highlighting this. We have now amended this in the text and also changed exit hole to the singular given there is only one exit hole in Figure 1c. **Changes made lines 155-157.**

(6) Spelling: symptom. **Comment on line 194 in the previously submitted manuscript.**
Response: We thank the reviewer for highlighting this and have now corrected this spelling. **Changes made line 208.**

(7) I suppose you need to have "sensitivities" and "specificities" (plural); otherwise there can be no covariances. Or does the use of singular work in English? You are native speakers, I am not. **Comment on lines 208, 209 in the previously submitted manuscript.**

Response: We thank the reviewer for this query. In this context we use the singular for sensitivity and specificity, because although there is more than one diagnostic test, there is one sensitivity and specificity value per diagnostic test. **No changes made.**

(8) I found myself looking for the very good prior knowledge of sensitivity and specificity (in purple colour) and did not find it. Can you add "(not shown in the figure)"? **Comment on line 302 in the previously submitted manuscript.**

Response: We thank the reviewer for highlighting this confusion. We have now added text to highlight that these distributions '*are not shown as separate figure panels because they are the same as the distributions that data were generated from (dashed lines).*' **Changes made lines 316-317.**

(9) I feel it is a little more standard to state that surveyor observations were "generated" from distributions rather than simulated. I make this remark because the word "simulated" is used in different contexts (understandably), e.g. also where you simulate sensitivity and specificity of individual assessors. In the latter context, I would find the word "simulate" more appropriate. But maybe that is an idiosyncratic preference that the authors don't share. Anyhow, If the terms used could be fine-tuned to the actual things you do it might be helpful to readers. **Comment on line 325 in the previously submitted manuscript.**
Response: We thank the reviewer for their comment. When we use the term simulated in the manuscript, we do so with the meaning that these datasets, surveyors and values are computer-generated using field informed statistical distributions to represent artificial surveyors. 'Generated' is the specific term for drawing samples from a distribution. Where we use the term 'simulated' this represents that these 'generated' values are from statistical distributions based on realistic field parameters to represent artificial surveyors/ survey datasets. We have been through the manuscript and amended phrasing accordingly to ensure clarity and consistency by either removing the term 'simulated' and/ or adding the term 'generated'. We now use the term 'generated' to specifically refer to the process of drawing samples from a given distribution. **Changes made lines 279; 280; 294; 311; 315; 317-318; 321-322; 351; 434; 452; 476; 507-508; 942; 963; 983; 1004; 1025; 1046; 1067; 1088; 1101-1103; 1115; 1133-1134; 1151-1152; 1172; 1190-1191.**

However, the text highlighted on line 325 in the original manuscript highlighted by this specific comment read '*Surveyor observations were simulated using 0.3 for lower true disease prevalence locations and 0.6 for higher true disease prevalence locations.*'. We retain use of the term simulated here given that we are not referring to the specific process of drawing samples from a specified distribution, but instead to a computer-generated dataset using field informed statistical distributions to represent artificial surveyors/ survey dataset at the different true disease prevalence sites. With this in mind, we have clarified the text to specify that we are referring to '*Surveyor observation datasets*'. **Change made line 340.**

(10) "using 0.3" -> "using a prevalence of 0.3" (I feel it would be much clearer). **Comment on line 325 in the previously submitted manuscript.**

Response: We thank the reviewer for highlighting this and have now amended this in the text for clarity. **Changes made lines 340-341.**

(11) add "and" at the end of the line. **Comment on line 391 in the previously submitted manuscript.**
Response: We thank the reviewer for highlighting this and have now amended this. **Change made line 408.**

(12) "values" -> "values of sensitivity and specificity" would be clearer (more explicit). **Comment on line 392 in the previously submitted manuscript.**

Response: We agree with the reviewer that this increases the clarity of the text and have amended the text accordingly. **Change made line 409**.

(13) Here you might use "expert data as a gold standard" or just "expert data as a standard"? **Comment on line 393 in the previously submitted manuscript.**

Response: We thank the reviewer for noting that the term gold-standard is missing, so we have added this to figure captions where missing **(changes made lines 409; 417; 931)**. We have also ensured that the term gold-standard is now consistently within single quotation marks throughout the manuscript to highlight that gold-standard assumes data has perfect sensitivity and specificity (defined at first mention in the manuscript) **(changes made lines 40; 107; 110-111; 122; 135; 275-276; 394; 524-525; 532; 552; 579; 609; 618-619)**.

(14) I suppose you mean here the sensitivity and specificity of EACH assessor, rather than the distribution of sensitivity and specificity in the WHOLE population of assessors. Please make it more explicit. **Comment on line 413 in the previously submitted manuscript.**

Response: We thank the reviewer for their suggestion and have now added text to improve clarity. **Change made line 430**.

(15) Did you mean here that the GENERATED specificity value of the assessor was not supported by the prior? More clarity is needed what is the meaning of "simulated". **Comment on line 417 in the previously submitted manuscript.**

Response: We thank the reviewer for highlighting this and have now removed the word simulated for clarity. We originally used the term 'simulated' here as an adjective to emphasise that the surveyor was an artificial surveyor, computer-generated using field informed statistical distributions. We acknowledge this is unclear in the context of the phrase '*the simulated surveyor specificity value*' and have now removed the term 'simulated'. Please refer to comment 9 for full details on use of the terms 'simulated' and 'generated' throughout the manuscript. **Change made line 434.**

(16) Maybe add "for same assessors" before the left bracket (. **Comment on line 424 in the previously submitted manuscript.**
Response: We thank the reviewer for their suggestion and have amended text to clarify this meaning for all relevant figure captions. **Changes made lines 440-441; 490-491; 944-945; 964-965; 985-986; 1006-1007; 1027-1028; 1048-1049; 1069-1070; 1090-1091; 1116-1117; 1135-1136; 1153-1154; 1173-1174; 1192-1193.**

(17) What is meant by "poor knowledge both symptoms"? Please write out your meaning for clarity. Isn't "poor knowledge both symptoms contradictory to "reliable knowledge on disease prevalence"? **Comment on line 424 (Fig 6 caption) in the previously submitted manuscript.**

Response: We thank the reviewer for highlighting this, and we have now amended the text in relevant figure captions throughout for clarity to highlight that we are referring to the knowledge of symptoms' sensitivity and specificity. **Changes made lines 441-442; 467; 491-492; 500; 933; 946; 955; 965-966; 974; 986-987; 995; 1008; 1017; 1028-1029; 1037; 1049-1050; 1058-1059; 1071; 1080; 1091-1092; 1106; 1117-1118; 1126; 1136-1137; 1144; 1154-1155; 1163; 1174-1175; 1183; 1193-1194.**

(18) “With this reliable prior knowledge” does not follow up from the preceding text (above Fig. 6. **Comment on line 432 in the previously submitted manuscript.**
Response: We thank the reviewer for highlighting this, and we have amended the text accordingly. **Change made line 450**.

(19) same comment as for Fig. 6 caption. **Comment on Fig. 7 caption (lines 448-449) in the previously submitted manuscript.**
Response: We thank the reviewer for their comments and we have amended this in the figure captions as outlined in response to comment 18.

(20) “which are more likely to be most effective spread wider across the landscape” Not clear. Check language/grammar. **Comment on line 527 in the previously submitted manuscript.**

Response: We thank the reviewer for highlighting that this is unclear. We have now amended the text for clarity and readability. **Changes made lines 545-547**.

(21) I think it was stated in the beginning of the paper that some of the citizen surveyors had been professional surveyors in the past. It may contradict this statement. **Comment on lines 551-552 in the previously submitted manuscript.**

Response: We thank the reviewer for highlighting this confusion. We have now added text to clarify that professional tree surveyors are not tree pest and disease professionals. These are distinct professions. **Changes made lines 171-172; 570-571**.

(22) The methodology is quite complex, and as noted by another reviewer, the code may not be particularly easy to use for non-experts, and I suppose that is an understatement. Hence, consider dropping “practical”. **Comment on line 599 in the previously submitted manuscript.**
Response: We thank the reviewer for their comment. We disagree that the approach outlined in this paper does not provide a practical solution to the problem of obtaining a gold-standard survey dataset. However, we have removed the word ‘practical’ to acknowledge that readers may disagree with this. **Change made line 618.**

(23) see comments on captions of Figs 6 and 7. “Poor sensitivity and specificity prior distributions. **Comment on Fig. 8 caption (lines 471-473) in the previously submitted manuscript.**

Response: We thank the reviewer for their comments and we have amended this in the figure captions as outlined in response to comment 18.
